# Bioprospecting Plant Growth Promoting Rhizobacteria for Enhancing the Biological Properties and Phytochemical Composition of Medicinally Important Crops

**DOI:** 10.3390/molecules27041407

**Published:** 2022-02-19

**Authors:** Asfa Rizvi, Bilal Ahmed, Mohammad Saghir Khan, Hossam S. El-Beltagi, Shahid Umar, Jintae Lee

**Affiliations:** 1 Department of Botany, School of Chemical and Life Sciences, Jamia Hamdard, Hamdard Nagar, New Delhi 110062, India; asfarizvi09@gmail.com (A.R.); sumer@jamiahamdard.ac.in (S.U.); 2 School of Chemical Engineering, Yeungnam University, 280 Daehak-Ro, Gyeongsan 38541, Korea; jtlee@ynu.ac.kr; 3 Department of Agricultural Microbiology, Faculty of Agricultural Sciences, Aligarh Muslim University, Aligarh 202002, India; khanms17@rediffmail.com; 4 Agricultural Biotechnology Department, College of Agriculture and Food Sciences, King Faisal University, P.O. Box 420, Al-Ahsa 31982, Saudi Arabia; 5 Biochemistry Department, Faculty of Agriculture, Cairo University, Gamma St., Cairo 12613, Egypt

**Keywords:** medicinal plants, plant growth promoting rhizobacteria, bioformulations, synergism, plant growth regulators, herbal medicines, secondary metabolites, bio-antimicrobials, abiotic stress, antioxidant enzymes

## Abstract

Traditionally, medicinal plants have long been used as a natural therapy. Plant-derived extracts or phytochemicals have been exploited as food additives and for curing many health-related ailments. The secondary metabolites produced by many plants have become an integral part of human health and have strengthened the value of plant extracts as herbal medicines. To fulfil the demand of health care systems, food and pharmaceutical industries, interest in the cultivation of precious medicinal plants to harvest bio-active compounds has increased considerably worldwide. To achieve maximum biomass and yield, growers generally apply chemical fertilizers which have detrimental impacts on the growth, development and phytoconstituents of such therapeutically important plants. Application of beneficial rhizosphere microbiota is an alternative strategy to enhance the production of valuable medicinal plants under both conventional and stressed conditions due to its low cost, environmentally friendly behaviour and non-destructive impact on fertility of soil, plants and human health. The microbiological approach improves plant growth by various direct and indirect mechanisms involving the abatement of various abiotic stresses. Given the negative impacts of fertilizers and multiple benefits of microbiological resources, the role of plant growth promoting rhizobacteria (PGPR) in the production of biomass and their impact on the quality of bio-active compounds (phytochemicals) and mitigation of abiotic stress to herbal plants have been described in this review. The PGPR based enhancement in the herbal products has potential for use as a low cost phytomedicine which can be used to improve health care systems.

## 1. Introduction

Medicinal plants, also called medicinal herbs, have been identified and herbal extracts have been used/practiced as traditional medicines since prehistoric times. Among herbal medicine practicing nations, India has a rich reserve of valuable medicinal plants due to its varying geographical conditions and climate diversity [1]. Almost all parts, including roots, stems, leaves and seeds, of medicinal plants have therapeutic potentials and can be used to treat different infections [2,3]. In addition, medicinal plants secrete several essential secondary metabolites or marker compounds having medicinal value that can be used for curing and preventing human illnesses [4,5,6]. Medicinal plants, in addition to their role in disease management, can also be used in cosmetics industries and other health care products to satisfy the user’s demands [7,8]. Due to this, medicinal plants are cultivated worldwide for both therapeutic purposes and to fulfil the demands of domestic industries. The World Health Organization (WHO) estimates that nearly 80% of the global population presently uses traditional herbal medicines for some aspect of primary health care [9]. In developing countries such as India, about 80% of the total population depends on conventional phytomedicines [10]. The traditional or classical medicines are widely used in many countries including India, China, Japan, Pakistan, Sri Lanka and Thailand. Thus, the interest in the development and recognition of the medicinal value of such an important group of plants in both industrialized and developing countries is increasing continuously [7]. The aromatic constituents and secondary metabolites such as flavonoids, polyphenols and other classes of phytochemicals are the two most important phytoconstituents that play important roles in treating various human diseases [11]. Due to this, the traditional herbal medicines are used throughout the world as an alternative to modern day medicines [12]. For instance, medicinal plants have been found effective in combating many human diseases including cancer [13] and other viral diseases such as AIDS, hepatitis, etc. [14,15,16]. The traditional plant-based medicines, therefore, occupy a significant place in the modern-day drug industries because they are safe, inexpensive and pharmacologically better compatible. In addition, the combination of various plant secondary metabolites has shown synergistic action and, hence, they are doubly effective [17]. Besides these, the compounds and metabolites secreted by herbal plant genotypes protect the producing plants from attack by microbes and insect pathogens [18,19].

Due to various therapeutic potentials and the presence of variable phytochemicals, medicinal plants hold a special significance in sustainable agriculture systems [20]. Therefore, the demand for cultivation of medicinal plants is steadily increasing worldwide. Hence, the focus has currently been shifted to the cultivation of different therapeutically useful medicinal plants such as ashwagandha, opium, aloe, isubgol, aonla, medicinal solanum, stevia, etc. using sustainable approaches [21]. To achieve these, chemical fertilization is practiced in the cultivation of herbal plants which, however, due to challenges such as cost and harmful impacts on the biomass, yield and valuable metabolites are avoided in medicinal plant production systems [22]. The application of low-cost natural resources and beneficial microbiological formulations (biofertilizers), especially those prepared from PGPR group organisms, either alone or in synergism with other agronomically beneficial microbiota, in this regard have been reported to optimize the production of good quality medicinal plants under both conventional and stressful conditions without depending on synthetic fertilizers [23,24,25]. Recently, the commercialization of various PGPR formulations has emerged as an inexpensive and non-hazardous microbiological tool to minimize the use of chemical input in the production of medicinal plants [26,27]. Acknowledging the importance of medicinal plants and herbal extracts in human health and the role of the microbiome in the yield optimization, the present review summarizes the current knowledge available on the mechanistic basis of PGPR in enhancing the quantity and quality of biomass, yield and bio-active compounds/phytochemicals of different important medicinal plants growing under normal and problem soils. The bio-antimicrobials secreted by different medicinal plants provide a low cost, ecofriendly and sustainable option to the public to improve health care systems.

## 2. Secondary Metabolites of Medicinal Plants: Importance for Human Health

Medicinal plants synthesize some specialized compounds or secondary metabolites, referred to as natural products, a rich source for modern pharmaceuticals and drug-based industries [28]. Such phytochemicals with therapeutic potentials play numerous important roles in maintaining the versatility of pharmacological effects. Based on their role in various metabolic processes, the phytochemicals have been grouped as primary and secondary metabolites. Of these, the primary metabolites affect basic physiological functions, whereas the secondary metabolites have the pharmacological effects. The secondary metabolites have also been found effective in disease management, which establishes their use in traditional medicines. In modern medicine, the secondary metabolites provide essential antimicrobial compounds needed for treating various diseases [29].

The bioactive compounds significantly influence the healthcare, food and cosmetics industries in terms of value addition and preservation [30,31]. The secondary metabolites play many crucial roles, for example, they: (i) act as a carbon resource for plants, (ii) defend plants against pathogen attack [32,33], (iii) impart colour to flowers which attracts pollinators and (iv) protect plants from harmful UV radiations [34]. Plant–environment interactions, their survival and propagation are largely dependent on the secondary metabolites of plants and, therefore, the plethora of different phytochemicals greatly affects the survival of plants [35,36]. Apart from these, the microbial and parasitic infections can effectively be cured by the extracts derived from several medicinal plants [37]. For instance, several antifungal proteins such as glucanase, chitinase, etc. and other non-enzymatic proteins have been found within the seeds of medicinal plants, which protects the developing embryo from many infections [38,39]. Shoemaker et al. [40] report in a study that over 400,000 species of plants exist on earth which possess huge reservoirs of bioactive compounds, but unfortunately, only a small fraction of these biomaterials has been explored for research studies. Following screening of several traditional medicinal plants, it was recorded that the plant-derived bioactive compounds exhibited a substantial number of therapeutic properties. Consequently, a large number of clinically important plant-derived compounds (bio-antimicrobials) are available today, which are used for the production of several antitumoural [41], antifungal [42,43] and anticancer drugs [44]. Due to the emergence of resistance among pathogens against various drugs and the adverse effects of modern medicines on human health, the medicinal plants and the compounds derived from them have received greater attention globally due both to their antimicrobial properties and almost total lack of side effects [45]. Recently, the essential oils of medicinal plants, for example, *Callistemon lanceolatus*, *Ocimum gratissimum*, *Cymbopogon winterianus* Jowit, *Cymbopogon flexuosus*, *Mentha longifolia* and *Vitex negundo* were reported to have variable inhibitory impacts on Gram positive (*Staphylococcus aureus*, *Micrococcus luteus* and *Bacillus subtilis)* and Gram negative *(Escherichia coli* and *Klebsiella pneumoniae)* bacteria, due to differences in their antioxidant and antimicrobial activities. Moreover, the essential oils increased the antibacterial and antioxidant efficiency of other compounds and caused a substantial decline in active doses when used in combination [46]. Furthermore, the phytocompounds, such as protocatechuic acid, 3,4-dihydroxyphenylacetic acid, (+)-catechin, chlorogenic acid, 4-hydroxybenzoic acid, caffeic acid, vanillic acid, syringic acid, 3-hydroxybenzoic acid, verbascoside, *p*-coumaric acid, ferulic acid, hesperidin, hyperoside and rosmarinic acid have been extracted from *Camellia sinensis*, *Erica arborea*, *Ilex paraguariensis*, *Rosemarinus officinalis* and *Thymus vulgaris* [47]. The extract derived from the rhizomes of rose plants, for example, acts as a stimulant. Rose root possesses many pharmacological and therapeutic properties. Salitroside tyrosol, cinnamic acid and glycosides (rosavin, rosarin, rosin) are some of the useful bio-active compounds derived from rose plants [48,49]. *Drosera* is yet another plant that produce various secondary metabolites, such as the most common naphthoquinones [50]. Similarly, the leaves and berries of bilberry (*Vaccinium myrtillus*) plants contain significant amounts of phenolic compounds [51]. Bilberries also secrete exceptionally high amounts of anthocyanins, which have strong antioxidant potential. The bilberry extract has shown promising results in the treatment of cancer and cardiovascular diseases. The flavonoids of bilberry plants also possess antimicrobial activities (antiviral, antibacterial and antifungal) and antiallergenic properties. Furthermore, the juice of cranberry can preclude urinary tract infections (UTI) in women [52]. The bio-efficacy of such metabolites, however, varies from plant genotype to genotype, source of extraction and chemical composition (Table 1).

## 3. Plant Growth Promoting Rhizobacteria Influencing the Cultivation of Medicinal Plants

The medicinal plant growers adopt various cultivation practices such as rotation of crops, use of varieties resistant to pathogens and chemical fertilizers, etc., to optimize their production [71]. Largely, the growers have focused on the conventional practices of fertilization while the scientific community or progressive farmers have shown interest in the use of bioformulations to optimize the safe production of phytochemicals and their medicinal value. However, due to the undesirable impact of chemical fertilizers and emergence of resistance among pathogens [72,73], there is an urgent need to reduce the use of agrochemicals in cultivation practices for augmenting the yield of medicinal plants and maintaining the quality of secondary metabolites. However, there are few studies which have reported the use of low cost and environmentally non-hazardous microbial communities in the production of medicinal plants and protection/alleviation of abiotic stresses [74,75]. Due to this, the use of economical, environmentally friendly and sustainable alternatives such as those of plant growth promoting rhizobacteria have been highlighted.

### 3.1. Direct Mechanisms

#### 3.1.1. Biological Nitrogen Fixation (BNF) and P-Solubilisation

Nitrogen, the most vital plant nutrient, can be supplied to plants by free living bacteria such as *Azotobacter*, associative bacterium *Azospirillum* or endophytic bacteria *Gluconoacetobacter diazotrophicus* [76,77]. Some of the other notable nitrogen-fixing PGPR are *Herbaspirillum*, *Bacillus*, *Burkholderia*, *Paenibacillus,* etc. [78]. Free-living nitrogen-fixing bacteria form a very close association with plants without penetrating the tissues and maintain sufficient numbers to supply available N to growing plants. Phosphorus, next only to N, is the other major plant nutrient which affects different growth and developmental processes such as photosynthesis, respiration, signal transduction and energy transfer of plants [79]. Various plant growth promoting rhizobacteria associated with insoluble P to soluble P transformation, and generally called phosphate solubilizing microorganisms (PSM), benefit plants by supplying P. The PSM inhabiting rhizospheres/phyllospheres/endospheres include bacteria, fungi and actinomycetes which, through different mechanisms, makes P available to plants [80,81]. The most widely accepted mechanism of P solubilisation by microbes in general includes the release of organic acids, OH^−^ ions, CO_2_ and protons. Some of the important P-solubilizing genera are *Pseudomonas* [82], *Bacillus* [83], *Burkholderia* [84], etc.

#### 3.1.2. Production of Phytohormones

Indoleacetic Acid, Cytokinins and Gibberellins

The phytohormone indole-3-acetic acid (IAA) is one of the major plant hormones excreted by more than 80% of the rhizosphere microbiomes [85,86], such as *Bacillus* sp. and *Enterobacter* sp. [87], which controls different physiological process of plants—(i) organogenesis, (ii) cellular activities such as growth, division and differentiation, (iii) gene regulation and (iv) responses to environmental variations for instance, light and gravity [88]. Moreover, the plants receiving IAA for extended periods have well-developed root systems which benefit the plants to absorb maximum nutrients and minerals from soils [89]. Cytokinins, another important phytohormone secreted by PGPR, are physiologically identical to IAA [78] and control cell division and the cell cycle in addition to activating many plant developmental processes [90]. The major stimulatory or inhibitory activity of cytokinin includes regulation of growth (root and shoot) and branching, control of shoot apical dominance, development of chloroplasts and controlling the relocation of nutrients from leaf to reproducing seeds [91]. In addition, cytokinin alters the size and activity of meristems through cell division activity of embryonic and mature plants [92]. Gibberellins (GAs) are the other group of important phytohormones that stimulate many metabolic events such as germination, flowering, stem elongation and fruit formation [93]. The synthesis of gibberellins among the microbiome is generally uncommon. However, species of *Bacillus*, for example, *B. licheniformis* and *B. amyloliquefaciens*, have been reported as gibberellin producers [94].

### 3.2. Indirect Mechanisms

#### 3.2.1. Aminocyclopropane-1-carboxylic Acid (ACC) Deaminase

ACC deaminase among plant growth modulators is a significant growth regulator that modifies the biochemical reactions and influences the development of plants indirectly by decreasing the level of stress hormone, ethylene [95,96]. A divergent group of plant growth promoting rhizobacteria secretes ACC deaminase which splits ACC into ammonia and α-ketobutyrate and thus limits its conversion to ethylene. Bacteria synthesizing ACC deaminase belongs to genera *Pseudomonas* [97], *Bacillus* [98], *Acinetobacter* [99], *Azospirillum* [100], *Achromobacter* [101], *Enterobacter* [102], *Burkholderia* [103], *Agrobacterium* [104], *Rhizobium* [105], *Serratia* [106], etc.

#### 3.2.2. Release of Siderophores, Cyanogenic Compounds and Ammonia

Siderophores are small molecular weight (≈200–2000 Daltons) compounds secreted by plant growth promoting bacteria [107,108], for example, *Bacillus* [109,110], *Pseudomonas* [111,112], etc., which can act as antagonists under iron-deficient conditions because they make iron inaccessible for uptake by harmful soil-borne phytopathogens. Siderophores thus control soil-borne phytopathogens by limiting iron availability [113] and, therefore, destroy their growth and disease-causing potential [114]. Among the different types of siderophores secreted by bacteria, pyoverdin, produced generally by pseudomonads [115], impedes the growth of phytopathogenic bacteria and fungi [116]. Pseudobactin siderophore, excreted by the *P. putida* strain B10, in contrast has been reported to destroy the growth and disease-causing ability of *Fusarium oxysporum* by restricting the iron supply. Collectively, the secretion of siderophores is one of the most important biological components of disease management and an indirect way of enhancing growth by siderophore positive PGPR. Apart from siderophores [117], different antibacterial compounds [118,119] produced by PGPR play important roles in preventing the damage caused by phytopathogens and concurrently augment the yield of crops.

The production of volatile cyanogenic substances such as hydrogen cyanide (HCN) through cyanogenesis by living organisms including PGPB [120,121] controls various plant diseases caused by bacterial or fungal phytopathogens, thereby assisting the plant growth promotion [122]. Mechanistically, HCN secreted generally by species of *Pseudomonas* [123] and *Bacillus* adversely affects the growth and spread of pathogenic microbes by inhibiting the transfer of electrons and supply of energy to the bacterial cells. For example, HCN positive bacterial strains have been found to have beneficial effects on seedling root growth of various plants by restricting the growth of phytopathogens [124]. In addition to producing cyanogens, many bacteria possess ammonia (NH_3_) secreting ability, which is also considered a unique plant growth enhancing feature [125,126].

#### 3.2.3. Secretion of Antibiotics and Lytic Enzymes

**Figure 1:**
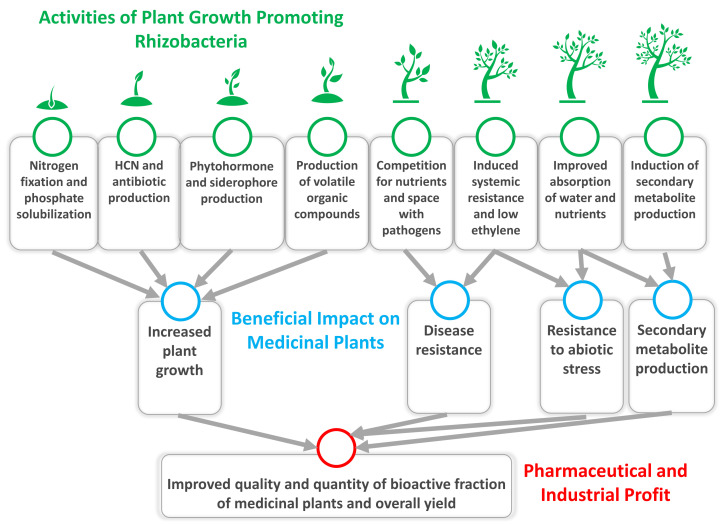
A schematic representation of direct (N_2_ fixation; P solubilisation; phytohormone secretion) and indirect (cyanogenesis, growth modulating enzymes, siderophores and induced systemic resistance, ISR, etc.) mechanisms adopted by plant growth promoting rhizobacteria for optimizing the growth, yield and quality of bio-antimicrobials of medicinal plants while growing under conventional or stressed environments at bench scale or under real field conditions.

Synthesis and release of antibiotics by PGPR is yet another important antagonistic trait which they use to manage the specific phytopathogens [127,128]. Studies by different workers have proved that the diffusible antibacterial drugs such as pyrrolnitrin, phycocyanin, 2,4-diacetylphloroglucinol (DAPG), etc. by different PGPR resulted in the suppression of phytopathogens [129,130]. Mechanistically, the antibiotics impair the integrity of the membranes and formation of initiation complexes on the small subunit of the ribosome [131]. As an example, the 2,4-diacetylphloroglucinol, an active and widely studied antibiotic produced by several strains of pseudomonas spp. has been found to damage the plasma membrane, alter vacuolization and disintegrate the cellular contents of *Pythium ultimum* var. *Sporangiiferum* sp., and it concurrently inhibited the zoospore formation [132]. Similarly, phenazine secreted by pseudomonas bacteria exhibits redox activity which can control the populations and infective abilities of *F. oxysporum* and *Gaeumannomyces graminis* [132]. Other important antibiotics are polymyxin, circulin and colistin, produced by strains of *Bacillus* which inhibits the growth of pathogenic bacteria and fungi [133]. Secretion of a series of cell wall degrading enzymes such as chitinases [134], glucanases [135], β-1,3-glucanases [136], cellulases [137], proteases [138], lipases [139], etc. by PGPR are the other factors that contribute in controlling the damage caused by microbial phytopathogens (Figure 1).

## 4. Rhizobacteria Mediated Improvement in Growth and Phytochemicals of Selected Medicinal Plants: Inoculation Effects

**Figure 2:**
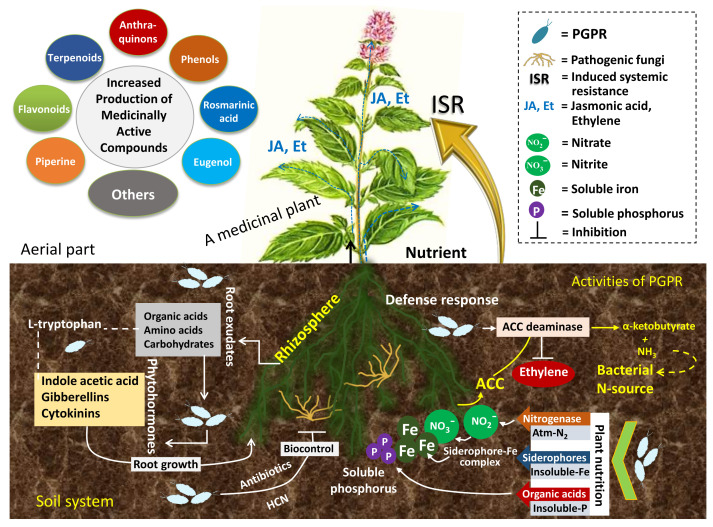
An overview of PGPR mediated nutrient mobilization, phytohormone supply and plant defence against microbial pathogens leading to the enhancement in the bio-chemical properties and yield of therapeutically bio-active compounds of medicinal plants growing under different environmental set up.

Although PGPR have shown tremendous increase in the biological and chemical features of many food and industrial crops [140,141,142], the data on the beneficial impact of PGPR on the performance of medicinal plants are relatively scarce [143,144,145] because very few greenhouse/field experiments targeting specifically PGPR–medicinal plant interactions have been conducted. Further, the mechanisms/pathways concerning the synthesis of secondary plant metabolites have not yet been fully understood, but the PGPR may also interact with medicinal plants in ways similar to those adopted by other crops. Therefore, an effort is directed herein towards understanding the role of PGPR in the growth and yield optimization of medicinal plants, citing some relevant examples. The inoculation of PGPR enhances the quality of the bioactive compounds of medicinal plants (Figure 2), which eventually benefits the pharmaceutical industries [146,147].

### 4.1. Datura

The genus *Datura*, often touted as “thorn apple”, belonging to the family Solanaceae with nine different species [148] has narcotic and medicinal values [149,150]. Additionally, ingestion of datura extracts or plant parts may cause severe poisoning or hallucinations [151]. The tropane alkaloids viz. hyoscyamine, atropine and scopolamine are the toxic components of datura [152,153]. Of these, atropine and scopolamine possess anticholinergic activities which are used in very low doses. Furthermore, scopolamine is an antimuscarinic agent and is a good muscle relaxant. It also possesses anti-nauseant properties and is a known antispasmodic agent which is used for treating motion sickness and in medication before operation [154]. Some of the other human health problems that may arise due to the ingestion of atropine includes blurred vision, reduced salivation, vasodilation and increased heart rate [155]. It can also be used as an antidote to organophosphorus insecticides [156]. Despite the impressive medicinal value and realization of the detrimental impact of chemical fertilization, few studies focusing on PGPR-Datura interactions have been reported [157,158]. The application of biofertilizer consisting of a mixture of nitrogen fixers (*Azotobacter chroococcum* and *Azospirillum brasilense*) and phosphate dissolving bacteria (*Bacillus megaterium* var. phosphaticum) along with mineral fertilizers (N and P) under field conditions demonstrated the best vegetative (plant height, number and total area and dry matter accumulation in leaves) and reproductive growth, mineral status, yield and quality of seeds and leaf anatomy [157,158]. In addition, PGPR enhanced the concentration of tropane alkaloids of thorn apple. Importantly, the combination of 50% NP mineral fertilizer and biofertilizer produced the best results compared to 100% NP mineral fertilizer when applied alone. Thus, the application of PGPR reduced the level of mineral fertilizer in datura cultivation, which is considered safer and more economical and can also minimize the environmental pollution caused by repeated application of mineral fertilizers [159]. To further strengthen the role of PGPR, Rahmoune et al. [147], in an independent greenhouse experiment, observed that the inoculation of beneficial PGPR such as *Pseudomonas plecoglossicida*, *Lysinibacillus fusiformis* and *Bacillus* sp. dramatically increased the growth and development of datura plants. Furthermore, the C/N ratio of plants was increased substantially. The alkaloid production in *D. stramonium* leaf, however, did not differ significantly, but its composition was altered in shoots following PGPR inoculation. Besides alkaloids, PGPR can also influence the composition and concentrations of amino acids. The amino acids and their derivatives detected in roots or in foliage of *D. stramonium* by gas chromatography–electron impact/Time-of-flight Mass spectrometry (GC–EI/TOF MS) included alanine, alanine 3-cyano-, alanine beta-, arginine, asparagine, aspartic acid, butanoic acid 4-amino-, butyro-1,4-lactam, glutamic acid, glutamine, glycine, histidine, homocysteine, homoserine, isoleucine, leucine, lysine, ornithine, ornithine-1,5-lactam, phenylalanine, proline, pyroglutamic acid, serine, threonine, tryptophan, tyrosine and valine [160].

### 4.2. Aloe vera

*Aloe vera* (*Aloe barbadensis* Miller) is a perennial plant belonging to family Liliaceae. The juice extracted from *A. vera* is effective in relieving digestive problems. *A. vera* also serves as a bio-antimicrobial and a skin protectant against harmful ultraviolet (UV) rays [161,162,163]. Other medicinal applications of *A. vera* include increased blood flow to wounded areas and enhanced production of fibroblasts. Certain liver related problems, gastric problems, inflammatory bowel disease, etc., can also be cured by *A. vera* [164]. In the form of gel, *A. vera* is used for topical (wounds, burns, skin irritations, etc.) as well as for internal applications (for treating constipation, coughs, ulcers, diabetes, headaches, arthritis, etc.) [165,166]. In order to optimize the biomass production and to improve phytochemical quality of *A. vera*, PGPR, either alone [167] or in combination with other biofertilizers such as PSB or mycorrhizal fungi, have been used [168]. For example, an increase in aloin content of *A. vera* following single or co-inoculation of rhizobacteria *Azospirillum*, *Azotobacter*, *Bacillus* and *Pseudomonas* has been reported [169]. Mamta et al. [170] in an identical experiment recorded a noteworthy upsurge in root length, leaf length and number of foliage, volume and dry weight of gel and whole plants when bio-primed with *Pseudomonas synxantha*, *Serratia marcescens*, *Burkholderia gladioli* and *Enterobacter hormaeche.* In addition, the aloin-A content of *A. vera* plants was increased due to PGPR application.

### 4.3. Withania somnifera (Ashwagandha)

*Withania somnifera* (Ashwagandha), a member of family Solanaceae, having several medicinal properties, has been used as a remedy in the ancient system of Indian medicine [171,172]. The underground (roots) parts of this plants are dried and are used to cure various nervous and sexual disorders. The drug obtained from Ashwagandha contains many biologically active compounds termed as ‘Withanolides’ [173]. Among various withanolides, Withaferin-A is therapeutically active and is found in the leaves and roots of plants. Withaferin-A has significant anticancer properties [174]. This plant can also be used as a potent anti-stress adaptogen, a potent antimicrobial agent and in the treatment of hypothyroidism [175,176]. The monumental medicinal value prompted Rajasekar and Elango [177] to grow this plant using a mixture of *Azospirillum*, *Azotobacter*, *Pseudomonas* and *Bacillus*. These PGPR either alone or in combination considerably augmented the height and length (root) of plants and alkaloid content of inoculated *W. somnifera* plants. In a follow up greenhouse production system, the growth and yield of *W. somnifera* was improved substantially following single or dual inoculation of *B. subtilis*, *P. fluorescens*, *A. chroococcum*, *A. brasilense*, *Methylobacterium radiotolerans*, *Exiguobacterium acetylicum*, *Paenibacillus polymyxa*, *Pantoea dispersa* and *B. sonorensis* [178]. The PGPR applications, in general, substantially enhanced the dry matter accumulation in root and shoot and whole plants, N and P in root and shoot, respectively, Withaferin-A concentration in roots and total withanolide content in plants relative to control. Among all PGPR, *B. sonorensis*, however, had significantly maximum beneficial impact on all the measured biological factors such as height, girth, root and shoot dry weight of plants and withanolide concentration [178]. The increase in the plants’ activity was attributed to inoculation of *B*. *sonorensis*, which possessed several PGP activities such as P solubilisation, IAA secretion, siderophore, amylase, ammonia and HCN production. These growth regulators together contributed to the maximum enhancement in the plant biomass and phytochemical production of *W. somnifera* plants [178].

### 4.4. Fenugreek

Fenugreek (*Trigonella foenum-graecum* L.) is a precious herbal plant which is spread worldwide. It belongs to the family Fabaceae and, apart from being grown as a medicinal plant [179,180], it is cultivated both as a spice and vegetable crop. The anti-atherosclerotic properties of fenugreek have been well documented [181]. Fenugreek also has some nutritional importance, for example, its seeds are full of compounds such as diosgenin, tannic acid, trigocoumarin, trigonelline, alkaloids, trigomethyl coumarin, gitogenin, vitamin A, etc. [64,182], whereas its foliage is rich in iron, calcium, *β*-carotene and other essential vitamins, which suggests that fenugreek has both nutritional and medicinal value [183].

Plant growth promoting rhizobacteria, when applied under cultivation practices, have displayed beneficial effects on the overall performance of medicinal plants. For this reason, Sharghi et al. [184] applied biofertilizers *Rhizobium meliloti*, *P. fluorescens* and a combination of both bacterial cultures to assess their impact on physiological and morphological characteristics of fenugreek cultivated under drought conditions. The results showed a significant increase in foliage area, fresh and dry biomass of shoot and root, P and K concentration, and water use efficacy (WUE) following inoculation with single or a mixture of PGPR. The individual PGPR formulation was, however, more effective compared to dual application while seed yield decreased in PGPR treated plants. In a follow up study, the co-inoculation impact of *Sinorhizobium meliloti* and *P. fluorescens* on the growth of fenugreek plants was evaluated under varying soil water levels. The *S. meliloti* and *P. fluorescens* applied together with 100% soil water level maximally increased the seed weight per plant. However, when the soil water level was 40%, the inoculated PGPR strains maximally enhanced the nicotinic acid and trigonelline of fenugreek plants. The applied PGPR strains showed positive/beneficial impacts on the morphological, physiological and phytochemical characteristics of fenugreek plants [185].

### 4.5. Turmeric

Turmeric (*Curcuma longa* L.), a rhizomatous herbaceous perennial plant, belongs to family Zingiberaceae. Turmeric is widely used as a spice crop, though it possesses tremendous medicinal value and, therefore, is widely used as a traditional or folk medicine [186]. Turmeric plants have been used to treat different diseases such as asthma, bronchial hyperactivity, rheumatism, diabetic wounds, sinusitis, smallpox, skin cancer, urinary tract infection and liver ailments, etc. [187]. Moreover, it has also been found effective against jaundice, abdominal pain, etc. [181]. The pharmacological traits such as anti-inflammatory, antioxidant, antimalarial, anticancer, hypolipidemic and immune-enhancer properties of turmeric are all attributed to curcumin, which is a yellow-coloured substance derived from the plant and is an essential ingredient of curcuminoids [182,183]. The chemical compound ‘curlone’ of turmeric has ROS scavenging (antioxidant) and antimutagenic activities [184].

Chemical fertilization such as with N, P and K is a common agronomic practice to optimize turmeric production worldwide [185,186]. The long term and random application of synthetic fertilizers, however, hampers the production of turmeric and alters the texture and nutrient pool of soil. Therefore, the use of PGPR across different physiological groups seems highly beneficial in the context of growth and yield of turmeric and the fertility of soil. In this regard, turmeric rhizomes interact with variously distributed soil microbial communities both as rhizospheres and as endophytes [187,188]. In general, the conventional rhizosphere microflora and microbial communities colonizing inside plant tissues (endophytic species) modulate the morpho-anatomical growth, secretion of secondary metabolites, curcumin content, antioxidants and biotic stress management [187,189,190,191,192,193,194,195]. The inoculation of turmeric plants with rhizobacterial cell suspensions containing *Pseudomonas* and *Bacillus* sp. (1:1) significantly enhanced the yield of rhizomes by 21%, plant height by 5% and weight of rhizome by 60% as compared to uninoculated controls [196]. Furthermore, a significant improvement in the curcumin content of *P. fluorescens* and *B. megaterium* inoculated turmeric plants has been reported [197]. Likewise, an increase in the number of leaves, height and biomass of shoot, dry biomass of rhizome and curcumin content of turmeric was recorded when plants were bioprimed with asymbiotic N_2_ fixer *A. chroococcum* [194]. Kumar et al. [198] also observed an identical increase in biological properties, yield attributes and curcumin content of *P. fluorescens* bacterized turmeric plants while antioxidant activity, flavanoids, phenol content and curcumin content were significantly enhanced when the rhizome of turmeric plants was inoculated with a dual culture of PGPR and AM-fungi [199].

### 4.6. Piper nigrum

Black pepper (*Piper nigrum* L.) is a commercial spice crop grown in different regions of the world. Both endophytic and biosensor bacteria can increase the growth and quality of black pepper [200]. The application of beneficial bacteria *B. tequilensis* strain NRRLB-41771 revealed a significantly greater root morphogenesis in black pepper when grown under greenhouse conditions. Moreover, an increment in the total N and P content in soil and plant tissues was recorded in rhizobacteria inoculated black pepper plants [201]. In addition, *Serratia nematodiphila* expressing P solubilisation activity, IAA secretion ability and siderophore excretion potential significantly influenced the growth of *P. nigrum* grown under greenhouse using sand: soil: FYM [202].

### 4.7. Basil

The genus *Ocimum* is a member of family Lamiaceae that contains over 150 cultivated aromatic perennial herb species, spread all over tropical and temperate regions [203]. *Ocimum* sp. is rich in essential oils consisting of various phenolic compounds and other natural products including polyphenols such as flavonoids and anthocyanins [204,205]. The leaves and flowering parts of sweet basil (*Ocimum basilicum*) have antispasmodic, aromatic, carminative and digestive properties [206]. Additionally, in various food preparations, basil is used as an ingredient in its fresh form, especially in Mediterranean cuisine. The essential oil derived from basil possesses antimicrobial [207] and insecticidal [208] properties while, due to its pleasant aroma, basil oil is used in food industries, pharmaceuticals, cosmetics, aromatherapy, etc. Considering the medicinal importance of basil and to facilitate the safe production of basil, Mangmang et al. [209] revealed that the *A. brasilense* inoculation increased the length, height and dry biomass of basil by 90%, 19%, and 44%, respectively, while plants also had more developed (25%) and bigger (61%) leaves. The bacterial inoculation altered the metabolic activities of inoculated plants and augmented the peroxidase activity and total concentration of *P. Basil* plants previously inoculated with bacterial strains and grown under an aquaponics system showed superior growth and upsurge in the leaf area, fresh herbage yield and root weight by 27, 11 and 11%, respectively. The increment in peroxidase activity (73%), IAA (27%) and protein contents (20%) following inoculation suggests that the PGPR, similar to other plants, could also serve as an important microbiological tool to facilitate the growth and yield stability of basil. Likewise, the *P. putida* strain 41, *A. chroococcum* strain 5 and *A. lipoferum* strain OF enhanced various biological and chemical parameters of basil plants such as dry and fresh weight of roots and shoots, height of shoots and N, P, K content. The essential oils content was also greatly enhanced relative to uninoculated plants [210].

### 4.8. Rosemary

Rosemary (*Rosmarinus officinalis* L.) belongs to family Labiatae and grows generally as a shrub or sometimes as herbaceous plants [211]. Morphologically, rosemary is an aromatic plant with dark green leaves and is cultivated mainly in the Mediterranean region. The leaves and flowering tops of rosemary plants are a rich source of flavonoids and phenolic acids, especially the rosmarinic acid and an essential oil which contains compounds such as pinene, camphene, cineole, borneol and camphor, all of which exhibit medicinal and stimulatory effects [212,213]. The oils extracted from rosemary have numerous therapeutic and antiseptic applications [214]; in addition, they are used in making soaps, perfumes, cosmetics, etc. They are also used in flavouring and preservation of foods [215]. Like many other plants, rosemary is also responsive to inoculations and has displayed improved yield following PGPR application [216]. The biological properties such as number of lateral of branches (43.95–46.39%), height of stem (29.04–38.57%), length of roots (32.31–37.14%), shoots (34.76–40.91%) and dry biomass of roots (62.89–70.70%) increased significantly due to inoculation of *B*. *subtilis*, *P*. *aeruginosa* and *Cedecia lapagei*. Additionally, the physiological features such as total chlorophyll, phenol and carotenoids of *R. officinalis* were augmented up to 31, 25 and 40% by bacterial inoculation relative to control. Besides this, a considerable improvement in plant nutrients (NPK) was also recorded [217]. The application of PGPR also significantly increased the essential oils of rosemary plants even while growing in salinized conditions [218]. The commercial biofertilizers *A. chroococcum* and *P. fluorescens,* when applied with humic and fulvic acids, had variable but significant impact on the number of stem, growth of plants, oil extract volume and plant height [219]. Studies by Kasmaei et al. [220] also revealed an obvious increment in dry biomass, nutrient mobilization, photosynthetic factors, carbohydrate, flavonoid and essential oils of rosemary by organic fertilizers, especially with composite application of PGPB and compost or biochar, but proline content declined in all treatments. This finding validated the positive impacts of PGPR, compost and boichar of *Azolla* on rosemary production and consequently increased the nutrient uptake and protected chlorophyll from degradation and enhanced the foliage quality [220].

### 4.9. Hyssopus

Hyssop (*Hyssopus officinalis*), which belongs to the family Lamiaceae [221], is one of the most important medicinal plant that produces essential oils and phenolic compounds. Phytoconstituents of this plant include quercetin-7-*O*-β-d-apiofuranosyl-(1→2)-β-d-xylopyranoside and quercetin-7-*O*-β-Dapiofuranosyl-(1→2)-β-d-xylopyranoside-3′-*O*-β-d-glucopyranoside. It also possesses antioxidant, anticonvulsant, antifungal, antimicrobial, antihemolytic, antiulcer, antispasmodic and many other medicinal properties [222]. The research conducted so far has shown that combination of organic materials and biofertilizers can be effective in enhancing the morphological features and productivity of hyssop. As an example, the organic manures (compost/vermicompost) and biofertilizer (*Azotobacter* and *Azospirillum*) used together had impressive positive influence on the biological and yield attributes, herb dry yield and concentration and yield of essential oil in *Hyssop* grown under field conditions [223]. Additionally, when *Hyssopus* plants were bacterized with PGPR, the growth regulators, plant metabolites and vitamins, etc. were increased which directly and significantly influenced the growth and development of plants. Among various rhizobacteria applied, *Azospirillum* demonstrated greatest valuable impact on the growth of *Hyssopus* plants, leading eventually to a substantial increase in all biological traits [224]. The beneficial impact of some PGPR on the overall growth and development of some medicinal plants is summarized in Table 2.

## 5. Abiotic Stress Alleviation in Medicinal Plants by Beneficial Rhizobacteria

Similar to many other plants, medicinal plants are also susceptible to various abiotic stresses such as drought, salinity, heavy metals, flooding, cold, nutrient deficiency [242,243] and biotic stresses [244] that have detrimental impact on growth, development and secretion of phytoconstituents. Findings indicate challenges akin to physiological imbalances such as overproduction of stress hormone, ethylene and nutritional imbalances that may influence the growth and therapeutic features of medicinal plants [245]. Reduction in the chlorophyll fluorescence (Fv/Fm) and relative water content (RWC) are some other harmful impacts on medicinal plants (e.g., *Mentha pulegium*) growing under stress. The antioxidant enzyme activity, lipid peroxidation, production of secondary metabolites and DPPH radical scavenging activity, however, increases in such valuable plants to combat stress. Application of rhizospheres/phyllospheres/endophytes PGPR can be useful in promoting the growth and yield stability while protecting the plants from harmful effects of single or multiple stress factors. In this context, inoculation of PGPR, *A. chroococcum* and *A. brasilense* substantially circumvented the lethal effects of drought stress and simultaneously improved the concentration of phytochemicals such as abcissic acid (ABA), soluble sugars, phenolic and flavonoids, etc. in pennyroyal plants even under severe drought stress conditions [227]. The alleviation of drought stress in *Glycyrrhiza uralensis*, a plant of extreme medicinal importance, by *B. pumilus* is also reported [34]. In this case, the chlorophyll content, photosynthetic rate and water state were improved in *B. pumilus* primed plants relative to non-inoculated plants, wherein the chloroplast membrane system was ruptured under water deficit conditions. The growth and survival rate of pepper plants was also enhanced following inoculation of IAA and ACC deaminase positive *B. licheniformis* strain K11 under drought stress [246]. The uninoculated pepper plants, however, did not survive 15 days after exposure to drought conditions, but the inoculated plants, on the contrary, survived for longer duration even under stress. A similar increase in the chlorophyll content and mineral uptake in sweet basil plants bacterized with *Pseudomonas* sp., *B. lentus* and *A. brasilense* under drought conditions is described [247]. Additionally, the PGPR inoculated *Hyoscyamus niger*, an important herbal plant which has high quantity of tropane alkaloids, demonstrated positive results. Inoculation with *P. putida* and *P. fluorescens* abated the toxic effects caused due to water scarcity in henbane plants and concurrently improved the growth and the content of tropane alkaloids [248].

**Figure 3:**
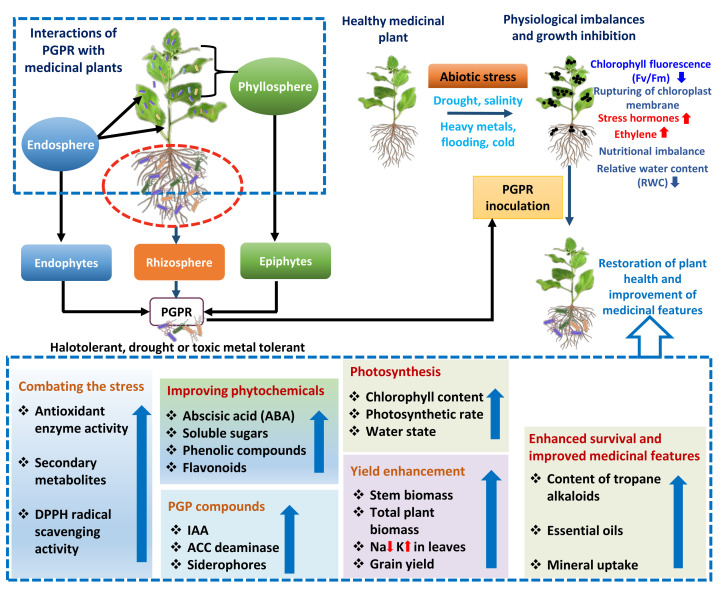
Various outcomes of endophytic, epiphytic and rhizospheric PGPR–medicinal plant interactions and management of abiotic stresses such as drought, salinity and metal toxicity resulting in the restoration of health of medicinal plants and overall enhancement in the biological growth, phytochemicals and bio-antimicrobials. PGPR = plant growth promoting rhizobacteria, IAA = indole-3-acetic acid, ACC = 1-aminocyclopropane-1-carboxylic acid (ACC).

Apart from drought stress, the performance of medicinal plants is also destructively influenced by high salt levels [249,250], which are though can be circumvented by PGPR inhabiting rhizospheres, phyllospheres or endospheres [245,251,252]. Recently, coriander seeds inoculated with composite cultures of *A. brasilense* and *A. chroococcum* grown with variable concentrations of salts displayed a noteworthy enhancement in the chlorophyll a and b content, grain yield, stem biomass and total plant biomass. Moreover, the dual inoculation significantly enhanced the antioxidant enzymes, CAT, but decreased the concentration of APX and GPX compared to uninoculated plants. A substantial decrease in Na but a considerable increase in K concentration in coriander foliage was also recorded in co-inoculated plants. Together, this finding can be used to solve coriander production problems under high salinity situations [253]. The interactions between PGPR and medicinal plants under various abiotic stresses are summarized in Figure 3.

Bidgoli et al. [218] showed that the yield of essential oil of *Rosmarinus officinalis* plants was enhanced when the plants were inoculated with rhizobacterial strain *P. fluorescens* over uninoculated plants, even under salinity stress conditions. The increment in the growth and yield of *Mentha arvensis* plants was recorded following inoculation with *Halomonas desiderata* and *Exiguobacterium oxidotolerans*, even under varying levels (100, 300 and 500 mM) of salts [254]. Generally, PGPR strains significantly increased the yield which, however, varied considerably with variation in salt concentrations. For instance, *H. desiderata* produced maximum yield at 100 and 300 mM NaCl, whereas *E. oxidotolerans* showed maximum yield at 500 mM NaCl concentration. Likewise, salt stress alleviation in medicinal plant *Sylebum marianum* and pepper following inoculation with halotolerant IAA positive *P. aureantiaca* strain TSAU22 and *P. extremorientalis* strain TSAU20 under saline conditions is reported [255,256]. Similar to salinity and drought stress, medicinal plants also suffer from toxicity of heavy metals which, however, can be relieved by the application of PGPR [257]. As an example, the siderophore-producing metal tolerant *Mesorhizobium panacihumi* strain DCY119T provided IAA to the plants, thereby promoting seedling growth and thus, eventually, conferring metal resistance to medicinal plant *Panax ginseng*. It was further established that *M. panacihumi* strain DCY119T could be employed for bioremediation purposes to improve the growth of ginseng plants in metal polluted soils [231].

## 6. Conclusions and Future Prospects

The pharmacological applications of medicinal plants depend largely on their secondary metabolites and bioactive phytochemicals. Due to the remarkable ability to influence human health positively, medicinal plants have become one of the most powerful and effective natural remedies in medical science. However, the cultivation of medicinal plants is severely threatened by abiotic stresses and injudicious applications of agrochemicals. The PGPR in this regard provides solutions to the problems of inexpensive and environmentally hazardous fertilizers that can enhance the overall growth, yield and quality of phytoconstituents of medicinal plants. In addition, stress tolerant PGPR also alleviate the various abiotic challenges and, in doing so, they allow the medicinal plants to grow efficiently even under a perturbed environment. Application of such beneficial microbes will provide four obvious benefits—(i) savings/reduction in fertilizers and pesticides input, (ii) protection of soil quality and biodiversity, (iii) restoration of declining herbal cultivation area and (iv) availability of natural medicines at reasonably low cost to every section of society and, therefore, the dramatic improvement in the human health systems. Therefore, PGPR mediated growth improvement of medicinal plants and yield optimization of bioactive phytochemicals opens up some new avenues in the development of low-cost herbal medicines, which may help the public during health care systems failures.

## Figures and Tables

**Table 1:** Secondary metabolites synthesized by some important medicinal plants.

S. No.	Medicinal Plant	Secondary Metabolites/Bioactive Compounds					Ref.
1.	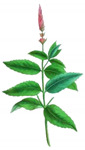 *Mentha piperita* (peppermint)	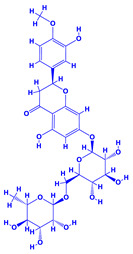 Hesperidin	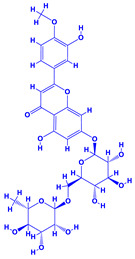 Diosmin	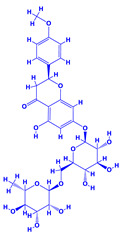 Didymin	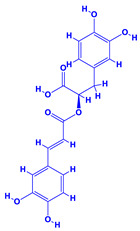 Rosmarinic acid		[53]
2.	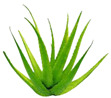 *Aloe vera* (aloe)	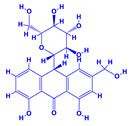 Aloin 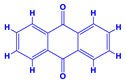 Anthraquinones	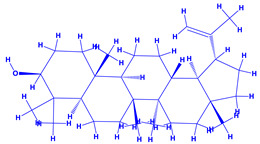 Lupeol 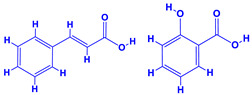 Cinnamic acid Salicylic acid		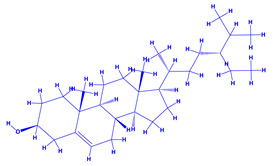 β-sitosterol or campestrol 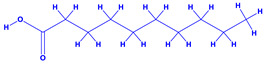 Fatty acids		[54,55]
3.	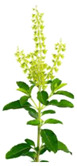 *Ocimum sanctum* (holy basil)	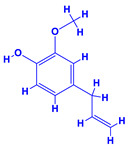 Eugenol	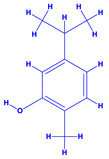 Carvacrol	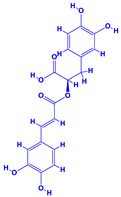 Rosmarinic acid	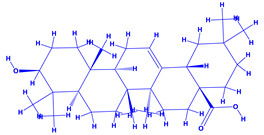 Oleanolic acid		[56]
4.	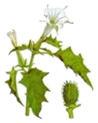 *Datura inoxia* (pricklyburr)	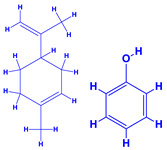 Terpenoids Phenols	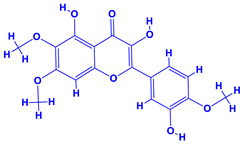 Flavonoids		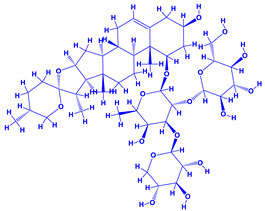 Saponins		[57]
5.	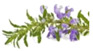 *Dracocephalum moldavica* (Moldavian balm or dragonhead)	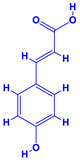 Phenolic acids (*p*-coumaric acid)	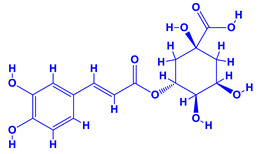 Chlorogenic acid		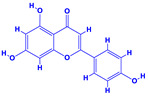 Ellagitannins (apigenin)	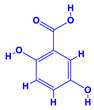 Flavonoids (gentisic acid)	[58]
6.	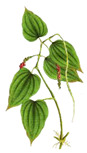 *Piper nigrum* (black pepper)	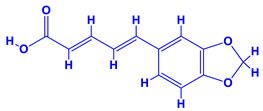 Piperic acid 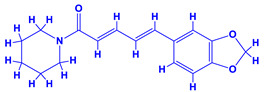 Piperine		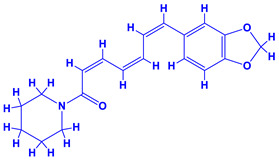 Piperttine 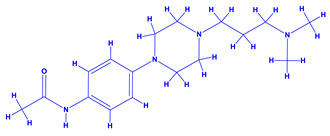 Piperamide			[59]
7.	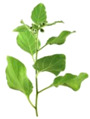 *Withania somnifera* (ashwagandha)	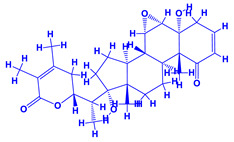 Withanine		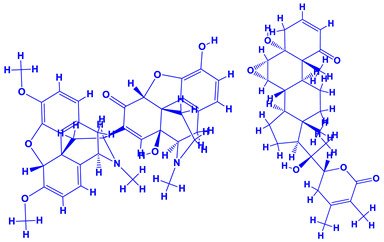 Somniferine Withanolides			[60,61]
8.	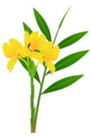 *Zingiber officinale* (ginger)	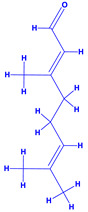 Geranial	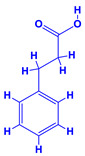 Phenylpropanoid	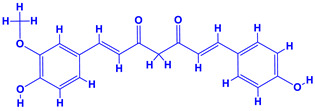 Oleoresin 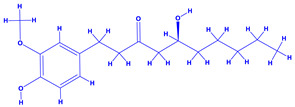 Gingerols			[62]
9.	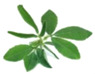 *Trigonella foenum-graecum* (fenugreek)	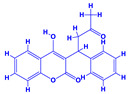 Coumarins 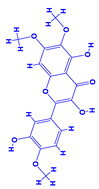 Flavonoids	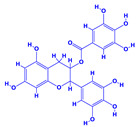 Polyphenols	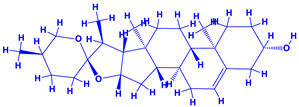 Steroidal sapogenins (diosgenin) 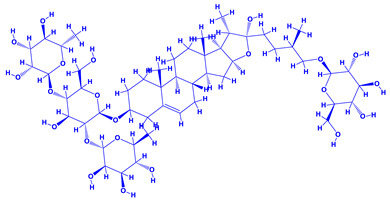 Alkaloids (protodioscin)			[63,64]
10.	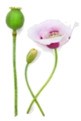 *Papaver Somniferum* (opium poppy)	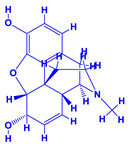 Morphine	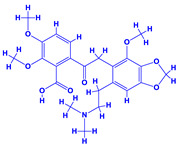 Narceine	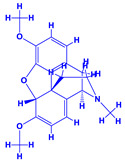 Thebaine	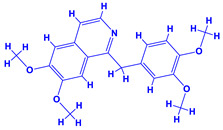 Papaverine		[65,66]
11.	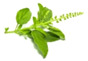 *Ocimum basilicum* (sweet basil)	Various phenolic compounds	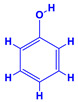	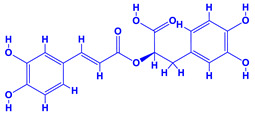			[67]
12.	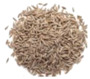 *Cuminum cyminum* (cumin)	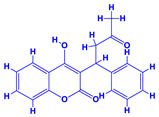 Coumarins	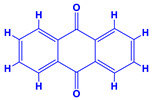 Anthraquinone	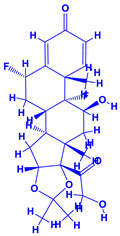 Steroids	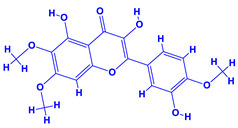 Flavonoids		[68]
13.	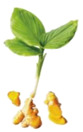 *Curcuma longa* (turmeric)	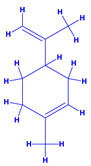 Terpenoids	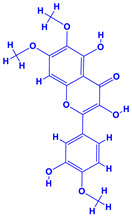 Flavonoids	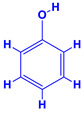 Phenols	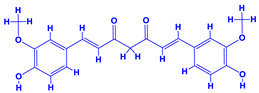 Curcumin		[69,70]

**Table 2:** Impact of plant growth promoting rhizobacteria on bio-chemical properties and yield of selected medicinal plants.

S. No.	Medicinal Plant	Bioinoculant Used	Bio-Chemical Traits	References
1.	*Limonium sinense*	*Bacillus flexus* KLBMP 4941	Enhanced synthesis of chlorophyll and flavonoids, osmotic regulation, increased activity of antioxidant enzymes and regulation of sodium/potassium homeostasis	[225]
2.	*Papaver somniferum*	*Pseudomonas putida* (WPTe)	Indole acetic acid production, growth and yield improvement, increased chlorophyll synthesis and stomatal conductance	[226]
3.	*Mentha pulegium* L.	*Azotobacter chroococcum* and *Azospirillum brasilense*	Mitigation of drought resistance, enhanced production of ascorbic acid, soluble sugars, proteins, flavonoids, total phenolics, oxygenated monoterpenes and 2,2-diphenyl-1-picrylhydrazyl (DPPH) scavenging activity	[227]
4.	*Foeniculum vulgare* sp. vulgare Mill	*Bacillus polymyxa*, *A. chroococcum*, and *Azospirillum lipoferum*	Increased plant attributes such as length, number of branches/plant, fresh weight of fruits and herbs, fruit yield, oil components, total phenolic content and photosynthesis	[228]
5.	*Dracocephalum moldavica*	*Micrococcus yunnanensis* and *Claroideoglomus etunicatum*	Enhanced photosynthesis, nutrient and dry matter accumulation, higher content of rosmarinic acid, eugenol, hesperetin and *p*-coumaric acid	[229]
6.	*Panax ginseng*	*Rhizobium panacihumi*	Biomass accumulation, higher proline levels, increased soluble sugar and total phenolic contents, scavenging response to oxidative stress, mitigation of aluminium stress	[230]
7.	*Panax ginseng* Meyer	*Mesorhizobium panacihumi* DCY119T	Production of siderophores, scavenging of ROS to circumvent iron stress, enhanced IAA production	[231]
8.	*Anoectochilus formosanus* (Wall.) Lindl. (YYB) and *Anoectochilus roxburghii* (Wall.) Lindl. (MRH)	*Bacillus velezensis* strains (ZJ-11 and D2WM)	Increased plant biological parameters such as height and weight, significant increase in flavonoid and kinsenoside content and reduction in the population of pathogenic fungi	[136]
9.	*Mentha piperita*	*Bacillus amyloliquefaciens* (GB03) and *Pseudomonas fluorescens* WCS417r	Bacterial inoculation improved various plant growth parameters including leaf number, surface area, and biomass, reduction in membrane lipid peroxidation and oxidative stress and increased phenolic content	[232]
10.	*Mentha piperita*	*Pseudomonas fluorescens* WCS417r, *P. putida* SJ04 and *B. subtilis* GB03	Rise in jasmonic acid (JA) and salicylic acid (SA) leading to glandular trichome density	[233]
11.	*Mentha piperita*	*Pantoea agglomerans* and *P. putida*	Phosphate solubilisation, enhanced biological attributes such as leaf number and length, number of stems, overall dry biomass accumulation and photosynthetic pigments	[234]
12.	*Mentha arvensis*	*Bacillus flexus* (Sd-30), *Stenotrophomonas* spp. (Az-30), and *Brevibacterium halotolerans* (Sd-6)	Enhanced photosynthesis, higher oil content and nutrient accumulation	[235]
13.	*Stevia rebaudiana*	PGPR strains (CA1001, CA2003 and CA2004)	IAA production	[236]
14.	*Mentha piperita*	*P. fluorescens*, *P. putida*, and *B. subtilis*	Enhanced emission of volatile organic compounds and phenolics	[237]
15.	*Ocimum basilicum* L.	Two biofertilizers containing *Pseudomonas* sp., *Azospirillum* sp., *Bacillus* sp., *Azotobacter* sp.	Increased dry matter accumulation in shoots and essential oils	[238]
16.	*Hyptis Suaveolens*	*Bacillus pumilus* and *Pseudomonas* *Pseudoalcaligenes*	Reduction of salinity impact, enhanced carotenoids and chlorophyll pigment	[239]
17.	*Handroanthus Ochraceus*	*Azospirillum brasilense*	Increased root volume, dry matter accumulation, enhanced density or size of glandular trichomes	[240]
18.	*Codonopsis pilosula* (Franch.) Nannf.	*Bacillus amyloliquefaciens* GB03	Two-fold increase in content of lobetyolin	[241]

## Data Availability

Not applicable.

## References

[1] Chowti P.S., Rudrapur S., Naik B.K. (2018). Production Scenario of Medicinal and Aromatic Crops in India. J. Pharmacogn. Phytochem..

[2] Sharifi-Rad J., Salehi B., Stojanović-Radić Z.Z., Fokou P.V.T., Sharifi-Rad M., Mahady G.B., Sharifi-Rad M., Masjedi M.-R., Lawal T.O., Ayatollahi S.A. (2020). Medicinal Plants Used in the Treatment of Tuberculosis-Ethnobotanical and Ethnopharmacological Approaches. Biotechnol. Adv..

[3] Mintah S.O., Asafo-Agyei T., Archer M.-A., Junior P.A.-A., Boamah D., Kumadoh D., Appiah A., Ocloo A., Boakye Y.D., Agyare C. (2019). Medicinal Plants for Treatment of Prevalent Diseases. Pharmacognosy—Medicinal Plants.

[4] Marques A.P.S., Bonfim F.P.G., Santos D.G.P.O., da Paz Lima M., Semir J., Martins E.R., Zucchi M.I., Hantao L.W., Sawaya A.C.H.F., Marques M.O.M. (2020). Chemical Diversity of Essential Oils from the Brazilian Medicinal Plant *Lychnophora Pinaster* Mart from Different Environments. Ind. Crops Prod..

[5] Ahmadi F., Samadi A., Rahimi A. (2020). Improving Growth Properties and Phytochemical Compounds of *Echinacea Purpurea* (L.) Medicinal Plant Using Novel Nitrogen Slow Release Fertilizer under Greenhouse Conditions. Sci. Rep..

[6] Ahmadi S.Z., Ghorbanpour M., Aghaee A., Hadian J. (2020). Deciphering Morpho-Physiological and Phytochemical Attributes of *Tanacetum Parthenium* L. Plants Exposed to C60 Fullerene and Salicylic Acid. Chemosphere.

[7] Sofowora A., Ogunbodede E., Onayade A. (2013). The Role and Place of Medicinal Plants in the Strategies for Disease Prevention. Afr. J. Tradit. Complement. Altern. Med..

[8] Lall N., Mahomoodally M.F., Esposito D., Steenkamp V., Zengin G., Steyn A., Oosthuizen C.B. (2020). Cosmeceuticals from Medicinal Plants. Front. Pharmacol..

[9] Shakya A.K. (2016). Medicinal Plants: Future Source of New Drugs. Int. J. Herb. Med..

[10] Kalauni D., Joshi A. (2018). Status of Medicinal and Aromatic Plant (Maps) and Socio-Economic Influence in Nepalese Livelihood-a Review Research. Acta Sci. Agric..

[11] Selamoglu Z. (2017). Polyphenolic Compounds in Human Health with Pharmacological Properties. J. Tradit. Med. Clin. Naturop..

[12] Selamoglu Z., Ustuntas H.E., Ozgen S. (2016). Traditional and Complementary Alternative Medicine Practices of Some Aromatic Plants in the Human Health. Res. J. Biol..

[13] Selamoglu Z. (2017). Biotechnological Approaches on Anticancer Activity of Flavonoids-Mini Review. Mod. Approaches Drug Des..

[14] Anywar G., Kakudidi E., Byamukama R., Mukonzo J., Schubert A., Oryem-Origa H., Jassoy C. (2021). A Review of the Toxicity and Phytochemistry of Medicinal Plant Species Used by Herbalists in Treating People Living with HIV/AIDS in Uganda. Front. Pharmacol..

[15] Omara T., Kiprop A.K., Ramkat R.C., Cherutoi J., Kagoya S., Moraa Nyangena D., Azeze Tebo T., Nteziyaremye P., Nyambura Karanja L., Jepchirchir A. (2020). Medicinal Plants Used in Traditional Management of Cancer in Uganda: A Review of Ethnobotanical Surveys, Phytochemistry, and Anticancer Studies. Evid.-Based Complement. Altern. Med..

[16] Matowa P.R., Gundidza M., Gwanzura L., Nhachi C.F.B. (2020). A Survey of Ethnomedicinal Plants Used to Treat Cancer by Traditional Medicine Practitioners in Zimbabwe. BMC Complement. Med. Ther..

[17] Dar R.A., Shahnawaz M., Qazi P.H. (2017). General Overview of Medicinal Plants: A Review. J. Phytopharm..

[18] Al-Ansari M.M., Andeejani A.M.I., Alnahmi E., AlMalki R.H., Masood A., Vijayaraghavan P., Rahman A.A., Choi K.C. (2021). Insecticidal, Antimicrobial and Antioxidant Activities of Essential Oil from *Lavandula latifolia* L. and Its Deterrent Effects on Euphoria Leucographa. Ind. Crops Prod..

[19] Iqrar I., Shinwari Z.K., El-Sayed A.S.A.F., Ali G.S. (2021). Exploration of Microbiome of Medicinally Important Plants as Biocontrol Agents against *Phytophthora parasitica*. Arch. Microbiol..

[20] Bhattacharjee T., Sen S., Chakraborty R., Maurya P.K., Chattopadhyay A. (2020). Cultivation of Medicinal Plants: Special Reference to Important Medicinal Plants of India. Herbal Medicine in India.

[21] Strzemski M., Dzida K., Dresler S., Sowa I., Kurzepa J., Szymczak G., Wójciak M. (2021). Nitrogen Fertilisation Decreases the Yield of Bioactive Compounds in *Carlina Acaulis* L. Grown in the Field. Ind. Crops Prod..

[22] Alipour A., Rahimi M.M., Hosseini S.M.A., Bahrani A. (2021). Mycorrhizal Fungi and Growth-Promoting Bacteria Improves Fennel Essential Oil Yield under Water Stress. Ind. Crops Prod..

[23] Nair R., Pandey S.K., Jyothsna J. (2021). Growth and Yield of Fenugreek (*Trigonella Foenum Graecum* L.) in Response to Different Levels of Phosphorus and Biofertilizer (Rhizobium and PSB) under Kymore Plateau and Satpura Hill Agro-Climatic Zone of Madhya Pradesh. Pharma Innov. J..

[24] Raiyani V.N., Kathiriya R.K., Thummer V.M., Rupareliya V.V. (2018). Effect of FYM and Biofertilizers on Growth, Yield Attributes and Yield of Fenugreek (*Trigonella Foenum-Graecum* L.). IJCS.

[25] Lobo C.B., Tomás M.S.J., Viruel E., Ferrero M.A., Lucca M.E. (2019). Development of Low-Cost Formulations of Plant Growth-Promoting Bacteria to Be Used as Inoculants in Beneficial Agricultural Technologies. Microbiol. Res..

[26] Karagöz F.P., Dursun A. (2019). Effects of Different PGPR Formulations, Chemical Fertilizers and Their Combinations on Some Plant Growth Characteristics of Poinsettia. Yüzüncü Yıl Üniversitesi Tarım Bilimleri Derg..

[27] Yang L., Yang C., Li C., Zhao Q., Liu L., Fang X., Chen X.-Y. (2016). Recent Advances in Biosynthesis of Bioactive Compounds in Traditional Chinese Medicinal Plants. Sci. Bull..

[28] Hussein R.A., El-Anssary A.A. (2019). Plants Secondary Metabolites: The Key Drivers of the Pharmacological Actions of Medicinal Plants. Herb. Med..

[29] Yilmaz M.A. (2020). Simultaneous Quantitative Screening of 53 Phytochemicals in 33 Species of Medicinal and Aromatic Plants: A Detailed, Robust and Comprehensive LC–MS/MS Method Validation. Ind. Crops Prod..

[30] Saha A., Basak B.B. (2020). Scope of Value Addition and Utilization of Residual Biomass from Medicinal and Aromatic Plants. Ind. Crops Prod..

[31] Leontopoulos S., Skenderidis P., Skoufogianni G. (2020). Potential Use of Medicinal Plants as Biological Crop Protection Agents. Biomed. J. Sci. Tech. Res.

[32] Atif M., Ilavenil S., Devanesan S., AlSalhi M.S., Choi K.C., Vijayaraghavan P., Alfuraydi A.A., Alanazi N.F. (2020). Essential Oils of Two Medicinal Plants and Protective Properties of Jack Fruits against the Spoilage Bacteria and Fungi. Ind. Crops Prod..

[33] Nunes A.R., Rodrigues A.L.M., de Queiróz D.B., Vieira I.G.P., Neto J.F.C., Junior J.T.C., Tintino S.R., de Morais S.M., Coutinho H.D.M. (2018). Photoprotective Potential of Medicinal Plants from Cerrado Biome (Brazil) in Relation to Phenolic Content and Antioxidant Activity. J. Photochem. Photobiol. B Biol..

[34] Zhang W., Xie Z., Zhang X., Lang D., Zhang X. (2019). Growth-Promoting Bacteria Alleviates Drought Stress of G. Uralensis through Improving Photosynthesis Characteristics and Water Status. J. Plant Interact..

[35] Zhang Y., Zheng L., Zheng Y., Xue S., Zhang J., Huang P., Zhao Y., Hao X., He Z., Hu Z. (2020). Insight into the Assembly of Root-Associated Microbiome in the Medicinal Plant *Polygonum cuspidatum*. Ind. Crops Prod..

[36] Singh A., Mishra A., Chaudhary R., Kumar V. (2020). Role of Herbal Plants in Prevention and Treatment of Parasitic Diseases. J. Sci. Res..

[37] Illamola S.M., Amaeze O.U., Krepkova L.V., Birnbaum A.K., Karanam A., Job K.M., Bortnikova V.V., Sherwin C.M.T., Enioutina E.Y. (2020). Use of Herbal Medicine by Pregnant Women: What Physicians Need to Know. Front. Pharmacol..

[38] Wu F., Yan M., Li Y., Chang S., Song X., Zhou Z., Gong W. (2003). CDNA Cloning, Expression, and Mutagenesis of a PR-10 Protein SPE-16 from the Seeds of *Pachyrrhizus erosus*. Biochem. Biophys. Res. Commun..

[39] Shoemaker M., Hamilton B., Dairkee S.H., Cohen I., Campbell M.J. (2005). In Vitro Anticancer Activity of Twelve Chinese Medicinal Herbs. Phytother. Res. Int. J. Devoted Pharmacol. Toxicol. Eval. Nat. Prod. Deriv..

[40] Albulescu M. (2015). Phytochemicals in Antitumor Herbs and Herbal Formulas. Phytochemicals-Isolation, Characterisation and Role in Human Health.

[41] Lu M., Li T., Wan J., Li X., Yuan L., Sun S. (2017). Antifungal Effects of Phytocompounds on Candida Species Alone and in Combination with Fluconazole. Int. J. Antimicrob. Agents.

[42] Ishaq M.S., Hussain M.M., Siddique Afridi M., Ali G., Khattak M., Ahmad S. (2014). In Vitro Phytochemical, Antibacterial, and Antifungal Activities of Leaf, Stem, and Root Extracts of Adiantum Capillus Veneris. Sci. World J..

[43] Ashraf M.A. (2020). Phytochemicals as Potential Anticancer Drugs: Time to Ponder Nature’s Bounty. BioMed Res. Int..

[44] Manandhar S., Luitel S., Dahal R.K. (2019). In Vitro Antimicrobial Activity of Some Medicinal Plants against Human Pathogenic Bacteria. J. Trop. Med..

[45] Sharma K., Guleria S., Razdan V.K., Babu V. (2020). Synergistic Antioxidant and Antimicrobial Activities of Essential Oils of Some Selected Medicinal Plants in Combination and with Synthetic Compounds. Ind. Crops Prod..

[46] Tlili N., Sarikurkcu C. (2020). Bioactive Compounds Profile, Enzyme Inhibitory and Antioxidant Activities of Water Extracts from Five Selected Medicinal Plants. Ind. Crops Prod..

[47] Verma A., Srivastava R., Sonar P.K., Yadav R. (2020). Traditional, Phytochemical, and Biological Aspects of *Rosa Alba* L.: A Systematic Review. Future J. Pharm. Sci..

[48] Boskabady M.H., Shafei M.N., Saberi Z., Amini S. (2011). Pharmacological Effects of *Rosa damascena*. Iran. J. Basic Med. Sci..

[49] Egan P.A., van der Kooy F. (2012). Coproduction and Ecological Significance of Naphthoquinones in Carnivorous Sundews (Drosera). Chem. Biodivers..

[50] Pires T.C.S.P., Caleja C., Santos-Buelga C., Barros L., Ferreira I.C.F.R. (2020). *Vaccinium myrtillus* L. Fruits as a Novel Source of Phenolic Compounds with Health Benefits and Industrial Applications-a Review. Curr. Pharm. Des..

[51] Hohtola A. (2010). Bioactive Compounds from Northern Plants. Bio-Farms for Nutraceuticals.

[52] Zhao B.T., Kim T.I., Kim Y.H., Kang J.S., Min B.S., Son J.K., Woo M.H. (2018). A Comparative Study of Mentha Arvensis L. and Mentha Haplocalyx Briq. by HPLC. Nat. Prod. Res..

[53] Rajeswari R., Umadevi M., Rahale C.S., Pushpa R., Selvavenkadesh S., Kumar K.S., Bhowmik D. (2012). Aloe Vera: Miracle Plant Its Medicinal and Traditional Uses in India. J. Pharmacogn. Phytochem..

[54] Maan A.A., Nazir A., Khan M.K.I., Ahmad T., Zia R., Murid M., Abrar M. (2018). The Therapeutic Properties and Applications of Aloe Vera: A Review. J. Herb. Med..

[55] Sawangjaroen N., Phongpaichit S., Subhadhirasakul S., Visutthi M., Srisuwan N., Thammapalerd N. (2006). The Anti-Amoebic Activity of Some Medicinal Plants Used by AIDS Patients in Southern Thailand. Parasitol. Res..

[56] Bagewadi Z.K., Muddapur U.M., Madiwal S.S., Mulla S.I., Khan A. (2019). Biochemical and Enzyme Inhibitory Attributes of Methanolic Leaf Extract of Datura Inoxia Mill. Environ. Sustain..

[57] Kamalizadeh M., Bihamta M., Zarei A. (2019). Drought Stress and TiO_2_ Nanoparticles Affect the Composition of Different Active Compounds in the Moldavian Dragonhead Plant. Acta Physiol. Plant..

[58] Takooree H., Aumeeruddy M.Z., Rengasamy K.R.R., Venugopala K.N., Jeewon R., Zengin G., Mahomoodally M.F. (2019). A Systematic Review on Black Pepper (*Piper Nigrum* L.): From Folk Uses to Pharmacological Applications. Crit. Rev. Food Sci. Nutr..

[59] Dhanani T., Shah S., Gajbhiye N.A., Kumar S. (2017). Effect of Extraction Methods on Yield, Phytochemical Constituents and Antioxidant Activity of *Withania somnifera*. Arab. J. Chem..

[60] Tripathi N., Shrivastava D., Ahmad Mir B., Kumar S., Govil S., Vahedi M., Bisen P.S. (2018). Metabolomic and Biotechnological Approaches to Determine Therapeutic Potential of *Withania Somnifera* (L.) Dunal: A Review. Phytomedicine.

[61] Mao Q.Q., Xu X.Y., Cao S.Y., Gan R.Y., Corke H., Beta T., Li H. (2019). Bin Bioactive Compounds and Bioactivities of Ginger (*Zingiber officinale* Roscoe). Foods.

[62] Chaudhary S., Chaudhary P.S., Chikara S.K., Sharma M.C., Iriti M. (2018). Review on Fenugreek (*Trigonella Foenum-Graecum* L.) and Its Important Secondary Metabolite Diosgenin. Not. Bot. Horti Agrobot..

[63] Wani S.A., Kumar P. (2018). Fenugreek: A Review on Its Nutraceutical Properties and Utilization in Various Food Products. J. Saudi Soc. Agric. Sci..

[64] Oh J.H., Ha I.J., Lee M.Y., Kim E.O., Park D., Lee J.H., Lee S.G., Kim D.W., Lee T.H., Lee E.J. (2018). Identification and Metabolite Profiling of Alkaloids in Aerial Parts of Papaver Rhoeas by Liquid Chromatography Coupled with Quadrupole Time-of-Flight Tandem Mass Spectrometry. J. Sep. Sci..

[65] Chandra De L. (2016). Bio-Diversity and Conservation of Medicinal and Aromatic Plants. Adv. Plants Agric. Res..

[66] Pirbalouti A.G., Malekpoor F., Salimi A., Golparvar A., Hamedi B. (2017). Effects of Foliar of the Application Chitosan and Reduced Irrigation on Essential Oil Yield, Total Phenol Content and Antioxidant Activity of Extracts from Green and Purple Basil. Acta Sci. Pol. Hortorum Cultus.

[67] Al-snafi A.E. (2017). The Pharmacological Activities of Cuminum Cyminum—A Review The Pharmacological Activities of Cuminum Cyminum—A Review Prof Dr Ali Esmail Al-Snafi. IOSR J. Pharm..

[68] Dutta B. (2015). Study of Secondary Metabolite Constituents and Curcumin Contents of Six Different Species of Genus Curcuma. J. Med. Plants Stud..

[69] Lee J., Jung Y., Shin J.H., Kim H.K., Moon B.C., Ryu D.H., Hwang G.S. (2014). Secondary Metabolite Profiling of Curcuma Species Grown at Different Locations Using GC/TOF and UPLC/Q-TOF MS. Molecules.

[70] Sharma T., Kaur A., Saajan S., Thakur R. (2020). Effect of Nitrogen on Growth and Yield of Medicinal Plants: A Review Paper. Eur. J. Mol. Clin. Med..

[71] Kole R.K., Roy K., Panja B.N., Sankarganesh E., Mandal T., Worede R.E. (2019). Use of Pesticides in Agriculture and Emergence of Resistant Pests. Indian J. Anim. Hlth.

[72] Siegwart M., Graillot B., Blachere Lopez C., Besse S., Bardin M., Nicot P.C., Lopez-Ferber M. (2015). Resistance to Bio-Insecticides or How to Enhance Their Sustainability: A Review. Front. Plant Sci..

[73] Köberl M., Schmidt R., Ramadan E.M., Bauer R., Berg G. (2013). The Microbiome of Medicinal Plants: Diversity and Importance for Plant Growth, Quality and Health. Front. Microbiol..

[74] Huang W., Long C., Lam E. (2018). Roles of Plant-Associated Microbiota in Traditional Herbal Medicine. Trends Plant Sci..

[75] Dent D., Cocking E. (2017). Establishing Symbiotic Nitrogen Fixation in Cereals and Other Non-Legume Crops: The Greener Nitrogen Revolution. Agric. Food Secur..

[76] Khairina Y., Jog R., Boonmak C., Toyama T., Oyama T., Morikawa M. (2021). Indigenous Bacteria, an Excellent Reservoir of Functional Plant Growth Promoters for Enhancing Duckweed Biomass Yield on Site. Chemosphere.

[77] Goswami D., Parmar S., Vaghela H., Dhandhukia P., Thakker J.N. (2015). Describing Paenibacillus Mucilaginosus Strain N3 as an Efficient Plant Growth Promoting Rhizobacteria (PGPR). Cogent Food Agric..

[78] Anand K., Kumari B., Mallick M.A. (2016). Phosphate Solubilizing Microbes: An Effective and Alternative Approach as Biofertilizers. J. Pharm. Pharm. Sci..

[79] Jeong S., Moon H.S., Nam K., Kim J.Y., Kim T.S. (2012). Application of Phosphate-Solubilizing Bacteria for Enhancing Bioavailability and Phytoextraction of Cadmium (Cd) from Polluted Soil. Chemosphere.

[80] Ghosh P., Rathinasabapathi B., Ma L.Q. (2015). Phosphorus Solubilization and Plant Growth Enhancement by Arsenic-Resistant Bacteria. Chemosphere.

[81] Paul D., Sinha S.N. (2017). Isolation and Characterization of Phosphate Solubilizing Bacterium Pseudomonas Aeruginosa KUPSB12 with Antibacterial Potential from River Ganga, India. Ann. Agrar. Sci..

[82] Saeid A., Prochownik E., Dobrowolska-Iwanek J. (2018). Phosphorus Solubilization by Bacillus Species. Molecules.

[83] Ghosh R., Barman S., Mukherjee R., Mandal N.C. (2016). Role of Phosphate Solubilizing Burkholderia Spp. for Successful Colonization and Growth Promotion of *Lycopodium Cernuum* L. (Lycopodiaceae) in Lateritic Belt of Birbhum District of West Bengal, India. Microbiol. Res..

[84] Myresiotis C.K., Vryzas Z., Papadopoulou-Mourkidou E. (2015). Effect of Specific Plant-growth-promoting Rhizobacteria (PGPR) on Growth and Uptake of Neonicotinoid Insecticide Thiamethoxam in Corn (*Zea Mays* L.) Seedlings. Pest Manag. Sci..

[85] Khalid A., Arshad M., Zahir Z.A. (2004). Screening Plant Growth-promoting Rhizobacteria for Improving Growth and Yield of Wheat. J. Appl. Microbiol..

[86] Saleem A.R., Brunetti C., Khalid A., Della Rocca G., Raio A., Emiliani G., De Carlo A., Mahmood T., Centritto M. (2018). Drought Response of *Mucuna Pruriens* (L.) DC. Inoculated with ACC Deaminase and IAA Producing Rhizobacteria. PLoS ONE.

[87] Carey N.S., Krogan N.T. (2017). The Role of AUXIN RESPONSE FACTOR s in the Development and Differential Growth of Inflorescence Stems. Plant Signal. Behav..

[88] Aeron A., Kumar S., Pandey P., Maheshwari D.K. (2011). Emerging Role of Plant Growth Promoting Rhizobacteria in Agrobiology. Bacteria in Agrobiology: Crop Ecosystems.

[89] Jha C.K., Saraf M. (2015). Plant Growth Promoting Rhizobacteria (PGPR). J. Agric. Res. Dev..

[90] Waldie T., Leyser O. (2018). Cytokinin Targets Auxin Transport to Promote Shoot Branching. Plant Physiol..

[91] Vatén A., Soyars C.L., Tarr P.T., Nimchuk Z.L., Bergmann D.C. (2018). Modulation of Asymmetric Division Diversity through Cytokinin and SPEECHLESS Regulatory Interactions in the Arabidopsis Stomatal Lineage. Dev. Cell.

[92] Martins A.O., Omena-Garcia R.P., Oliveira F.S., Silva W.A., Hajirezaei M.-R., Vallarino J.G., Ribeiro D.M., Fernie A.R., Nunes-Nesi A., Araújo W.L. (2019). Differential Root and Shoot Responses in the Metabolism of Tomato Plants Exhibiting Reduced Levels of Gibberellin. Environ. Exp. Bot..

[93] Shahzad R., Waqas M., Khan A.L., Asaf S., Khan M.A., Kang S.-M., Yun B.-W., Lee I.-J. (2016). Seed-Borne Endophytic Bacillus Amyloliquefaciens RWL-1 Produces Gibberellins and Regulates Endogenous Phytohormones of Oryza Sativa. Plant Physiol. Biochem..

[94] Magnucka E.G., Pietr S.J. (2015). Various Effects of Fluorescent Bacteria of the Genus Pseudomonas Containing ACC Deaminase on Wheat Seedling Growth. Microbiol. Res..

[95] Glick B.R. (2014). Bacteria with ACC Deaminase Can Promote Plant Growth and Help to Feed the World. Microbiol. Res..

[96] Orozco-Mosqueda M., Duan J., DiBernardo M., Zetter E., Campos-García J., Glick B.R., Santoyo G. (2019). The Production of ACC Deaminase and Trehalose by the Plant Growth Promoting Bacterium Pseudomonas Sp. UW4 Synergistically Protect Tomato Plants against Salt Stress. Front. Microbiol..

[97] Misra S., Chauhan P.S. (2020). ACC Deaminase-Producing Rhizosphere Competent Bacillus Spp. Mitigate Salt Stress and Promote Zea Mays Growth by Modulating Ethylene Metabolism. 3 Biotech.

[98] Daur I., Saad M.M., Eida A.A., Ahmad S., Shah Z.H., Ihsan M.Z., Muhammad Y., Sohrab S.S., Hirt H. (2018). Boosting Alfalfa (*Medicago Sativa* L.) Production with Rhizobacteria from Various Plants in Saudi Arabia. Front. Microbiol..

[99] García J.E., Maroniche G., Creus C., Suárez-Rodríguez R., Ramirez-Trujillo J.A., Groppa M.D. (2017). In Vitro PGPR Properties and Osmotic Tolerance of Different Azospirillum Native Strains and Their Effects on Growth of Maize under Drought Stress. Microbiol. Res..

[100] Ali S., Kim W.-C. (2018). Plant Growth Promotion under Water: Decrease of Waterlogging-Induced ACC and Ethylene Levels by ACC Deaminase-Producing Bacteria. Front. Microbiol..

[101] Sarkar A., Ghosh P.K., Pramanik K., Mitra S., Soren T., Pandey S., Mondal M.H., Maiti T.K. (2018). A Halotolerant Enterobacter Sp. Displaying ACC Deaminase Activity Promotes Rice Seedling Growth under Salt Stress. Res. Microbiol..

[102] Tagele S.B., Kim S.W., Lee H.G., Lee Y.S. (2019). Potential of Novel Sequence Type of Burkholderia Cenocepacia for Biological Control of Root Rot of Maize (*Zea mays* L.) Caused by Fusarium Temperatum. Int. J. Mol. Sci..

[103] Malambane G., Nonaka S., Shiba H., Ezura H., Tsujimoto H., Akashi K. (2018). Comparative Effects of Ethylene Inhibitors on Agrobacterium-Mediated Transformation of Drought-Tolerant Wild Watermelon. Biosci. Biotechnol. Biochem..

[104] Kumar P., Dubey R.C., Maheshwari D.K., Bajpai V. (2016). ACC Deaminase Producing Rhizobium Leguminosarum Rpn5 Isolated from Root Nodules of *Phaseolus Vulgaris* L.. Bangladesh J. Bot..

[105] Maxton S.P., Prasad S.M., Andy A., Masih S.A. (2017). Characterization of ACC Deaminase Producing B. Cepacia, C. Freundii and S. Marcescens for Plant Growth Promoting Activity. Int. J. Curr. Microbiol. Appl. Sci..

[106] Devi R., Thakur R. (2018). Screening and Identification of Bacteria for Plant Growth Promoting Traits from Termite Mound Soil. J. Pharm. Phytochem..

[107] Gupta G., Parihar S.S., Ahirwar N.K., Snehi S.K., Singh V. (2015). Plant Growth Promoting Rhizobacteria (PGPR): Current and Future Prospects for Development of Sustainable Agriculture. J. Microb. Biochem. Technol..

[108] Ghazy N., El-Nahrawy S. (2021). Siderophore Production by Bacillus Subtilis MF497446 and Pseudomonas Koreensis MG209738 and Their Efficacy in Controlling Cephalosporium Maydis in Maize Plant. Arch. Microbiol..

[109] Bharucha U.D., Patel K.C., Trivedi U.B. (2013). Antifungal Activity of Catecholate Type Siderophore Produced by Bacillus Sp.. Int. J. Res. Pharm. Sci..

[110] Vindeirinho J.M., Soares H.M.V.M., Soares E.V. (2021). Modulation of Siderophore Production by Pseudomonas Fluorescens through the Manipulation of the Culture Medium Composition. Appl. Biochem. Biotechnol..

[111] Luján A.M., Gómez P., Buckling A. (2015). Siderophore Cooperation of the Bacterium Pseudomonas Fluorescens in Soil. Biol. Lett..

[112] Bhat M.A., Rasool R., Ramzan S. (2019). Plant Growth Promoting Rhizobacteria (PGPR) for Sustainable and Eco-Friendly Agriculture. Acta Sci. Agric..

[113] Meena M., Swapnil P., Zehra A., Aamir M., Dubey M.K., Patel C.B., Upadhyay R.S. (2019). Virulence Factors and Their Associated Genes in Microbes. New and Future Developments in Microbial Biotechnology and Bioengineering.

[114] Peek M.E., Bhatnagar A., McCarty N.A., Zughaier S.M. (2012). Pyoverdine, the Major Siderophore in Pseudomonas Aeruginosa, Evades NGAL Recognition. Interdiscip. Perspect. Infect. Dis..

[115] Ruiz J.A., Bernar E.M., Jung K. (2015). Production of Siderophores Increases Resistance to Fusaric Acid in Pseudomonas Protegens Pf-5. PLoS ONE.

[116] Beneduzi A., Ambrosini A., Passaglia L.M.P. (2012). Plant Growth-Promoting Rhizobacteria (PGPR): Their Potential as Antagonists and Biocontrol Agents. Genet. Mol. Biol..

[117] Sureshbabu K., Amaresan N., Kumar K. (2016). Amazing Multiple Function Properties of Plant Growth Promoting Rhizobacteria in the Rhizosphere Soil. Int. J. Curr. Microbiol. Appl. Sci..

[118] Sivasakthi S., Usharani G., Saranraj P. (2014). Biocontrol Potentiality of Plant Growth Promoting Bacteria (PGPR)-Pseudomonas Fluorescens and Bacillus Subtilis: A Review. Afr. J. Agric. Res..

[119] Rijavec T., Lapanje A. (2017). Cyanogenic *Pseudomonas* Spp. Strains Are Concentrated in the Rhizosphere of Alpine Pioneer Plants. Microbiol. Res..

[120] Lukkani N.J., Reddy E.C.S. (2014). Evaluation of Plant Growth Promoting Attributes and Biocontrol Potential of Native Fluorescent *Pseudomonas* Spp. against Aspergillus Niger Causing Collar Rot of Ground Nut. Int. J. Plant Anim. Environ. Sci..

[121] Fouzia A., Allaoua S., Hafsa C.-S., Mostefa G. (2015). Plant Growth Promoting and Antagonistic Traits of Indigenous Fluorescent *Pseudomonas* Spp. Isolated from Wheat Rhizosphere and A. Halimus Endosphere. Eur. Sci. J..

[122] Reetha A.K., Pavani S.L., Mohan S. (2014). Hydrogen Cyanide Production Ability by Bacterial Antagonist and Their Antibiotics Inhibition Potential on Macrophomina Phaseolina (Tassi.) Goid. Int. J. Curr. Microbiol. Appl. Sci..

[123] Singh M., Singh D., Gupta A., Pandey K.D., Singh P.K., Kumar A. (2019). Plant Growth Promoting Rhizobacteria: Application in Biofertilizers and Biocontrol of Phytopathogens. PGPR Amelioration in Sustainable Agriculture.

[124] Ahmad E., Zaidi A., Khan M.S. (2016). Effects of Plant Growth Promoting Rhizobacteria on the Performance of Greengram under Field Conditions. Jordan J. Biol. Sci..

[125] Ahmad E., Khan M.S., Zaidi A. (2013). ACC Deaminase Producing Pseudomonas Putida Strain PSE3 and Rhizobium Leguminosarum Strain RP2 in Synergism Improves Growth, Nodulation and Yield of Pea Grown in Alluvial Soils. Symbiosis.

[126] Tariq M., Noman M., Ahmed T., Hameed A., Manzoor N., Zafar M. (2017). Antagonistic Features Displayed by Plant Growth Promoting Rhizobacteria (PGPR): A Review. J. Plant Sci. Phytopathol..

[127] Wang H., Tang X., Wang H., Shao H.B. (2015). Proline Accumulation and Metabolism-Related Genes Expression Profiles in Kosteletzkya Virginica Seedlings under Salt Stress. Front. Plant Sci..

[128] Liu K., McInroy J.A., Hu C.-H., Kloepper J.W. (2018). Mixtures of Plant-Growth-Promoting Rhizobacteria Enhance Biological Control of Multiple Plant Diseases and Plant-Growth Promotion in the Presence of Pathogens. Plant Dis..

[129] Meyer S.L.F., Everts K.L., Gardener B.M., Masler E.P., Abdelnabby H.M.E., Skantar A.M. (2016). Assessment of DAPG-Producing *Pseudomonas fluorescens* for Management of *Meloidogyne incognita* and *Fusarium oxysporum* on Watermelon. J. Nematol..

[130] Maksimov I.V., Abizgil’Dina R.R., Pusenkova L.I. (2011). Plant Growth Promoting Rhizobacteria as Alternative to Chemical Crop Protectors from Pathogens. Appl. Biochem. Microbiol..

[131] De Souza J.T., Arnould C., Deulvot C., Lemanceau P., Gianinazzi-Pearson V., Raaijmakers J.M. (2003). Effect of 2, 4-Diacetylphloroglucinol on Pythium: Cellular Responses and Variation in Sensitivity among Propagules and Species. Phytopathology.

[132] Chin-A-Woeng T.F.C., Bloemberg G.V., Lugtenberg B.J.J. (2003). Phenazines and Their Role in Biocontrol by Pseudomonas Bacteria. New Phytol..

[133] Srivastava S., Bist V., Srivastava S., Singh P.C., Trivedi P.K., Asif M.H., Chauhan P.S., Nautiyal C.S. (2016). Unraveling Aspects of Bacillus Amyloliquefaciens Mediated Enhanced Production of Rice under Biotic Stress of *Rhizoctonia solani*. Front. Plant Sci..

[134] Figueroa-López A.M., Cordero-Ramírez J.D., Martínez-Álvarez J.C., López-Meyer M., Lizárraga-Sánchez G.J., Félix-Gastélum R., Castro-Martínez C., Maldonado-Mendoza I.E. (2016). Rhizospheric Bacteria of Maize with Potential for Biocontrol of Fusarium Verticillioides. SpringerPlus.

[135] El-Gamal N.G., Shehata A.N., Hamed E.R., Shehata H.S. (2016). Improvement of Lytic Enzymes Producing Pseudomonas Fluorescens and Bacillus Subtilis Isolates for Enhancing Their Biocontrol Potential against Root Rot Disease in Tomato Plants. Res. J. Pharm. Biol. Chem. Sci..

[136] Ashwini N., Srividya S. (2014). Potentiality of Bacillus Subtilis as Biocontrol Agent for Management of Anthracnose Disease of Chilli Caused by Colletotrichum Gloeosporioides OGC1. 3 Biotech.

[137] Illakkiam D., Anuj N.L., Ponraj P., Shankar M., Rajendhran J., Gunasekaran P. (2013). Proteolytic Enzyme Mediated Antagonistic Potential of *Pseudomonas Aeruginosa* against *Macrophomina phaseolina*. Indian J. Exp. Biol..

[138] Melkamu T., Diriba M., Gezahegn B., Girma A. (2013). Antagonistic Effects of Rhizobacteria against Coffee Wilt Disease Caused by *Gibberella xylarioides*. Asian J. Plant Pathol..

[139] Mekonnen H., Kibret M. (2021). The Roles of Plant Growth Promoting Rhizobacteria in Sustainable Vegetable Production in Ethiopia. Chem. Biol. Technol. Agric..

[140] Dos Santos R.M., Diaz P.A.E., Lobo L.L.B., Rigobelo E.C. (2020). Use of Plant Growth-Promoting Rhizobacteria in Maize and Sugarcane: Characteristics and Applications. Front. Sustain. Food Syst..

[141] Riahi L., Cherif H., Miladi S., Neifar M., Bejaoui B., Chouchane H., Masmoudi A.S., Cherif A. (2020). Use of Plant Growth Promoting Bacteria as an Efficient Biotechnological Tool to Enhance the Biomass and Secondary Metabolites Production of the Industrial Crop Pelargonium Graveolens L’Hér. under Semi-Controlled Conditions. Ind. Crops Prod..

[142] Wei M., Zhang M., Huang G., Yuan Y., Fu C., Yu L. (2020). Coculture with Two Bacillus Velezensis Strains Enhances the Growth of Anoectochilus Plants via Promoting Nutrient Assimilation and Regulating Rhizosphere Microbial Community. Ind. Crops Prod..

[143] Wang M., Bian Z., Shi J., Wu Y., Yu X., Yang Y., Ni H., Chen H., Bian X., Li T. (2020). Effect of the Nitrogen-Fixing Bacterium Pseudomonas Protegens CHA0-ΔretS-Nif on Garlic Growth under Different Field Conditions. Ind. Crops Prod..

[144] Sharma S., Singh V., Kumar V., Devi S., Shukla K.P., Tiwari A., Singh J., Bisht S. (2015). Plant Growth-Promoting Rhizobacteria (PGPR): Emergence and Future Facets in Medicinal Plants. Plant-Growth-Promoting Rhizobacteria (PGPR) and Medicinal Plants.

[145] Chakrabartty I., Kalita N.K., Boruah P., Katiyar V., Hakeem K.R., Rangan L. (2020). Physico-Rheological Characterization of Organically Derived Seed Samples from Alpinia Nigra (Gaertn.) BL Burtt, an Ethnic Medicinal Plant of Northeast India. Ind. Crops Prod..

[146] Rahmoune B., Morsli A., Khelifi-Slaoui M., Khelifi L., Strueh A., Erban A., Kopka J., Prell J., van Dongen J.T. (2017). Isolation and Characterization of Three New PGPR and Their Effects on the Growth of Arabidopsis and Datura Plants. J. Plant Interact..

[147] Geeta R., Gharaibeh W. (2007). Historical Evidence for a Pre-Columbian Presence of Datura in the Old World and Implications for a First Millennium Transfer from the New World. J. Biosci..

[148] Srivastava R., Srivastava P. (2020). The Medicinal Significance of Datura Stramonium-A Review. Biomed. J. Sci. Tech. Res..

[149] Khan J., Khan R., Qureshi R.A. (2013). Ethnobotanical Study of Commonly Used Weeds of District Bannu, Khyber Pakhtunkhwa (Pakistan). J. Med. Plants Stud..

[150] Trancă S.D., Szabo R., Cociş M. (2017). Acute Poisoning Due to Ingestion of Datura Stramonium–a Case Report. Rom. J. Anaesth. Intensive Care.

[151] Li J., Lin B., Wang G., Gao H., Qin M. (2012). Chemical Constituents of Datura Stramonium Seeds. Zhongguo Zhong Yao Za Zhi= Zhongguo Zhongyao Zazhi China J. Chin. Mater. Med..

[152] Berkov S., Zayed R., Doncheva T. (2006). Alkaloid Patterns in Some Varieties of Datura Stramonium. Fitoterapia.

[153] Van Wyk B.-E., Van Heerden F., Van Oudtshoorn B. (2002). Poisonous Plants of South Africa.

[154] Ivancheva S., Nikolova M., Tsvetkova R. (2006). Pharmacological Activities and Biologically Active Compounds of Bulgarian Medicinal Plants. Phytochem. Adv. Res..

[155] Kumral N.A., Çobanoğlu S., Yalcin C. (2010). Acaricidal, Repellent and Oviposition Deterrent Activities of *Datura Stramonium* L. against Adult *Tetranychus Urticae* (Koch). J. Pest Sci..

[156] Rahmoune B., Zerrouk I.Z., Morsli A., Khelifi-Slaoui M., Khelifi L., Do Amarante L. (2017). Phenylpropanoids and Fatty Acids Levels in Roots and Leaves of *Datura stramonium* and *Datura innoxia*. Int. J. Pharm. Pharm. Sci..

[157] Božić D., Jovanović L., Raičević V., Pavlović D., Sarić-Krsmanović M., Vrbničanin S. (2014). The Effect of Plant Growth Promoting Rhizobacteria on *Datura Stramonium* L., *Abutilon Theophrasti* Med., *Onopordon Acanthium* L. and *Verbascum Thapsus* L. Seed Germination. Pestic. I Fitomed..

[158] Nassar R.M.A., Boghdady M.S., Selim D.A. (2015). Effect of Mineral and Bio-Fertilizers on Vegetative Growth, Mineral Status, Seed Yield, Tropane Alkaloids and Leaf Anatomy of Thorn Apple Plant (*Datura Stramonium* L.). Middle East J. Agric. Res.

[159] Rahmoune B., Zerrouk I.Z., Bouzaa S., Morsli A., Khelifi-Slaoui M., Ludwig-Müller J., Khelifi L. (2019). Amino Acids Profiling in Datura Stramonium and Study of Their Variations after Inoculation with Plant Growth Promoting Rhizobacteria. Biologia.

[160] Kahramanoğlu İ., Chen C., Chen J., Wan C. (2019). Chemical Constituents, Antimicrobial Activity, and Food Preservative Characteristics of Aloe Vera Gel. Agronomy.

[161] Waithaka P.N., Gathuru E.M., Githaiga B.M., Kazungu R.Z. (2018). Antimicrobial Properties of Aloe Vera, Aloe Volkensii and Aloe Secundiflora from Egerton University. Acta Sci. Microbiol..

[162] Kumar S., Jakhar D.S., Singh R. (2017). Evaluating Antimicrobial Activity of Aloe Vera Plant Extract in Human Life. Biomed. J. Sci. Tech. Res..

[163] Meena N.K., Tara N., Saharan B.S. (2018). Review on PGPR: An Alternative for Chemical Fertilizers to Promote Growth in Aloe Vera Plants. Int. J. Curr. Microbiol. Appl. Sci..

[164] Oryan A., Mohammadalipour A., Moshiri A., Tabandeh M.R. (2016). Topical Application of Aloe Vera Accelerated Wound Healing, Modeling, and Remodeling: An Experimental Study. Ann. Plast. Surg..

[165] Sahu P.K., Giri D.D., Singh R., Pandey P., Gupta S., Shrivastava A.K., Kumar A., Pandey K.D. (2013). Therapeutic and Medicinal Uses of Aloe Vera: A Review. Pharmacol. Pharm..

[166] Meena N., Saharan B.S. (2017). Effective Biocontrol of Leaf Rot Disease on Aloe Vera Plant by PGPR in Green House Experiment. BEPLS.

[167] Khajeeyan R., Salehi A., Dehnavi M.M., Farajee H., Kohanmoo M.A. (2021). Growth Parameters, Water Productivity and Aloin Content of Aloe Vera Affected by Mycorrhiza and PGPR Application under Different Irrigation Regimes. S. Afr. J. Bot..

[168] Kalaimani S., Kandeepan D.R.C. (2018). Effect of rhizobacterial inoculation on alkaloid aloin content of aloevera. IJRAR—Int. J. Res. Anal. Reviews..

[169] Mamta G., Rahi P., Pathania V., Gulati A., Singh B., Bhanwra R.K., Tewari R. (2012). Comparative Efficiency of Phosphate-Solubilizing Bacteria under Greenhouse Conditions for Promoting Growth and Aloin-A Content of Aloe Barbadensis. Arch. Agron. Soil Sci..

[170] Pandey V., Ansari W.A., Misra P., Atri N. (2017). Withania Somnifera: Advances and Implementation of Molecular and Tissue Culture Techniques to Enhance Its Application. Front. Plant Sci..

[171] Umadevi M., Rajeswari R., Rahale C.S., Selvavenkadesh S., Pushpa R., Kumar K.P.S., Bhowmik D. (2012). Traditional and Medicinal Uses of Withania Somnifera. Pharma Innov..

[172] Aslam S., Raja N.I., Hussain M., Iqbal M., Ejaz M., Ashfaq D., Fatima H., Shah M.A., Ehsan M. (2017). Current Status of *Withania Somnifera* (L.) Dunal: An Endangered Medicinal Plant from Himalaya. Am. J. Plant Sci..

[173] Davis L., Kuttan G. (2002). Effect of Withania Somnifera on CTL Activity. J. Exp. Clin. Cancer Res. CR.

[174] Ali N.A., Jülich W.-D., Kusnick C., Lindequist U. (2001). Screening of Yemeni Medicinal Plants for Antibacterial and Cytotoxic Activities. J. Ethnopharmacol..

[175] Panda S., Kar A. (1999). Withania Somnifera and Bauhinia Purpurea in the Regulation of Circulating Thyroid Hormone Concentrations in Female Mice. J. Ethnopharmacol..

[176] Rajasekar S., Elango R. (2011). Effect of Microbial Consortium on Plant Growth and Improvement of Alkaloid Content in *Withania Somnifera* (Ashwagandha). Curr. Bot..

[177] Anuroopa N., Bagyaraj D.J. (2017). Selection of an Efficient Plant Growth Promoting Rhizobacteria for Inoculating Withania Somnifera. J. Sci. Ind. Res..

[178] Zandi P., Basu S.K., Cetzal-Ix W., Kordrostami M., Chalaras S.K., Khatibai L.B. (2017). Fenugreek (*Trigonella Foenum-graecum* L.): An Important Medicinal and Aromatic Crop. Active Ingredients from Aromatic and Medicinal Plants.

[179] Fernández-Aparicio M., Emeran A.A., Rubiales D. (2008). Control of *Orobanche Crenata* in Legumes Intercropped with Fenugreek (*Trigonella Foenum-Graecum*). Crop Prot..

[180] Syed Q.A., Rashid Z., Ahmad M.H., Shukat R., Ishaq A., Muhammad N., Rahman H.U.U. (2020). Nutritional and Therapeutic Properties of Fenugreek (*Trigonella foenum-graecum*): A Review. Int. J. Food Prop..

[181] Warke V.B., Deshmukh T.A., Patil V.R. (2011). Development and Validation of RP-HPLC Method for Estimation of Diosgenin in Pharmaceutical Dosage Form. Asian J. Pharm. Clin. Res..

[182] Danesh T.S., Mehrafarin A., Naghdi B.H., Khalighi S.F. (2014). Changes in Growth and Trigonelline/Mucilage Production of Fenugreek (*Trigonella Foenum-Graecum* L.) under Plant Growth Regulators Application. J. Med. Plants.

[183] Sharghi A., Bolandnazar S., Badi H.N., Mehrafarin A., Sarikhani M.R. (2017). The Effects of Plant Growth Promoting Rhizobacteria (PGPR) on Growth Characteristics of Fenugreek under Water Deficit Stress. Adv. Biores..

[184] Sharghi A., Badi H.N., Bolandnazar S., Mehrafarin A., Sarikhani M.R. (2018). Morphophysiological and Phytochemical Responses of Fenugreek to Plant Growth Promoting Rhizobacteria (PGPR) under Different Soil Water Levels. Folia Hortic..

[185] Amalraj A., Pius A., Gopi S., Gopi S. (2017). Biological Activities of Curcuminoids, Other Biomolecules from Turmeric and Their Derivatives–A Review. J. Tradit. Complement. Med..

[186] Kumar A., Singh A.K., Kaushik M.S., Mishra S.K., Raj P., Singh P.K., Pandey K.D. (2017). Interaction of Turmeric (*Curcuma Longa* L.) with Beneficial Microbes: A Review. 3 Biotech.

[187] Bundy R., Walker A.F., Middleton R.W., Booth J. (2004). Turmeric Extract May Improve Irritable Bowel Syndrome Symptomology in Otherwise Healthy Adults: A Pilot Study. J. Altern. Complement. Med..

[188] Panahi Y., Saadat A., Beiraghdar F., Nouzari S.M.H., Jalalian H.R., Sahebkar A. (2014). Antioxidant Effects of Bioavailability-Enhanced Curcuminoids in Patients with Solid Tumors: A Randomized Double-Blind Placebo-Controlled Trial. J. Funct. Foods.

[189] Mukerjee A., Vishwanatha J.K. (2009). Formulation, Characterization and Evaluation of Curcumin-Loaded PLGA Nanospheres for Cancer Therapy. Anticancer Res..

[190] Osorio-Tobón J.F., Carvalho P.I.N., Barbero G.F., Nogueira G.C., Rostagno M.A., de Almeida Meireles M.A. (2016). Fast Analysis of Curcuminoids from Turmeric (*Curcuma longa* L.) by High-Performance Liquid Chromatography Using a Fused-Core Column. Food Chem..

[191] Qazi M.A., Iqbal M.N., Ashraf M., Khan N.I., Ahmad R., Umar F., Naeem U., Mughal K.M., Khalid M., Iqbal Z. (2020). NPK requirements of turmeric for cultivation in Punjab. Pak. J. Sci..

[192] Ojikpong T.O. (2018). Effect of Planting Dates and NPK (15: 15: 15) Fertilizer on the Growth an Yield of Turmeric (Curcuma Longa Linn). Int. J. Agric. Environ. Sci..

[193] Vinayarani G., Prakash H. (2018). Growth Promoting Rhizospheric and Endophytic Bacteria from Curcuma Longa L. as Biocontrol Agents against Rhizome Rot and Leaf Blight Diseases. Plant Pathol. J..

[194] Kumar A., Singh R., Giri D.D., Singh P.K., Pandey K.D. (2014). Effect of Azotobacter Chroococcum CL13 Inoculation on Growth and Curcumin Content of Turmeric (*Curcuma Longa* L.). Int. J. Curr. Microbiol. Appl. Sci..

[195] Prabhukarthikeyan S.R., Keerthana U., Raguchander T. (2018). Antibiotic-Producing Pseudomonas Fluorescens Mediates Rhizome Rot Disease Resistance and Promotes Plant Growth in Turmeric Plants. Microbiol. Res..

[196] Suryadevara N., Ponmurugan P. (2012). Response of Turmeric to Plant Growth Promoting Rhizobacteria (Pgpr) Inoculation under Different Levels of Nitrogen. Int. J. Biol. Technol..

[197] Boominathan U., Sivakumaar P.K. (2012). A Liquid Chromatography Method for the Determination of Curcumin in PGPR Inoculated Curcuma Longa. L Plant. Int. J. Pharm. Sci. Res..

[198] Kumar A., Singh M., Singh P.P., Singh S.K., Singh P.K., Pandey K.D. (2016). Isolation of Plant Growth Promoting Rhizobacteria and Their Impact on Growth and Curcumin Content in Curcuma Longa L.. Biocatal. Agric. Biotechnol..

[199] Dutta S.C., Neog B. (2016). Accumulation of Secondary Metabolites in Response to Antioxidant Activity of Turmeric Rhizomes Co-Inoculated with Native Arbuscular Mycorrhizal Fungi and Plant Growth Promoting Rhizobacteria. Sci. Hortic..

[200] Shobha M.S. (2018). Effect of Endophytic and Plant Growth Promoting Rhizobacteria against Foot Rot Disease of *Piper Nigrum* L.. Int. J. Environ. Agric. Biotechnol..

[201] Dastager S.G., Deepa C.K., Pandey A. (2011). Growth Enhancement of Black Pepper (*Piper Nigrum*) by a Newly Isolated Bacillus Tequilensis NII-0943. Biologia.

[202] Pandey A., Deepa C.K., Dastager S.G. (2011). Potential Plant Growth-Promoting Activity of Serratia Nematodiphila NII-0928 on Black Pepper (*Piper Nigrum* L.). World J. Microbiol. Biotechnol..

[203] Tangpao T., Chung H.-H., Sommano S.R. (2018). Aromatic Profiles of Essential Oils from Five Commonly Used Thai Basils. Foods.

[204] Balanescu F., Mihaila M.D.I., Cârâc G., Furdui B., Vînătoru C., Avramescu S.M., Lisa E.L., Cudalbeanu M., Dinica R.M. (2020). Flavonoid Profiles of Two New Approved Romanian Ocimum Hybrids. Molecules.

[205] Mousavi L., Salleh R.M., Murugaiyah V. (2018). Phytochemical and Bioactive Compounds Identification of Ocimum Tenuiflorum Leaves of Methanol Extract and Its Fraction with an Anti-Diabetic Potential. Int. J. Food Prop..

[206] Ahmed A.F., Attia F.A.K., Liu Z., Li C., Wei J., Kang W. (2019). Antioxidant Activity and Total Phenolic Content of Essential Oils and Extracts of Sweet Basil (*Ocimum basilicum* L.) Plants. Food Sci. Hum. Wellness.

[207] Mittal R., Kumar R., Chahal H.S. (2018). Antimicrobial Activity of Ocimum Sanctum Leaves Extracts and Oil. J. Drug Deliv. Ther..

[208] Rodríguez-González Á., Álvarez-García S., González-López Ó., Da Silva F., Casquero P.A. (2019). Insecticidal Properties of *Ocimum basilicum* and *Cymbopogon winterianus* against *Acanthoscelides obtectus*, Insect Pest of the Common Bean (*Phaseolus vulgaris*, L.). Insects.

[209] Mangmang J.S., Deaker R., Rogers G. (2016). Inoculation Effect of *Azospirillum Brasilense* on Basil Grown under Aquaponics Production System. Org. Agric..

[210] Ordookhani K., Sharafzadeh S., Zare M. (2011). Influence of PGPR on Growth, Essential Oil and Nutrients Uptake of Sweet Basil. Adv. Environ. Biol..

[211] Atikbekkara F., Bousmaha L., Talebbendiab S.A., Boti J.B., Casanova J. (2007). Chemical Composition of Essential Oil of *Rosmarinus Officinalis* L. Grown in the Tlemcen Region. J. Biol. Health.

[212] Nieto G., Ros G., Castillo J. (2018). Antioxidant and Antimicrobial Properties of Rosemary (*Rosmarinus officinalis*, L.): A Review. Medicines.

[213] Singletary K. (2016). Rosemary: An Overview of Potential Health Benefits. Nutr. Today.

[214] Habtemariam S. (2016). The Therapeutic Potential of Rosemary (*Rosmarinus Officinalis*) Diterpenes for Alzheimer’s Disease. Evid.-Based Complement. Altern. Med..

[215] Arnold N., Valentini G., Bellomaria B., Hocine L. (1997). Comparative Study of the Essential Oils from *Rosmarinus Eriocalyx* Jordan & Fourr. from Algeria and *R. Officinalis* L. from Other Countries. J. Essent. Oil Res..

[216] Dehghani Bidgoli R., Ghiaci Yekta M. (2020). Investigating the Effect of Plant Growth-Promoting Rhizobacteria (*Pseudomonas Putida* R112) on Essential Oil of *Rosmarinus Officinalis* L. under Irrigation of Urban Purified Wastewater. J. Med. Plants.

[217] Sharma M., Sood G., Chauhan A. (2021). Bioprospecting Beneficial Endophytic Bacterial Communities Associated with Rosmarinus Officinalis for Sustaining Plant Health and Productivity. World J. Microbiol. Biotechnol..

[218] Bidgoli R.D., Azarnezhad N., Akhbari M., Ghorbani M. (2019). Salinity Stress and PGPR Effects on Essential Oil Changes in *Rosmarinus Officinalis* L.. Agric. Food Secur..

[219] Cáceres J.A., Cuervo A.J.L., Rodríguez C.J.L. (2017). Effect of Organic Fertilization on Yield and Quality of Rosemary (*Rosmarinus officinalis* L.) Essential Oil. Agron. Colomb..

[220] Kasmaei L.S., Yasrebi J., Zarei M., Ronaghi A., Chasemi R., Saharkhiz M.J., Ahmadabadi Z., Schnug E. (2019). Impacts of PGPR, Compost and Biochar of Azolla on Dry Matter Yield, Nutrient Uptake, Physiological Parameters and Essential Oil of *Rosmarinus Officinalis* L.. J. Kult..

[221] Omidbaigi R. (2005). Production and Processing of Medicinal Plants. Beh-Nashr Mashhad.

[222] Tahir M., Khushtar M., Fahad M., Rahman M.A. (2018). Phytochemistry and Pharmacological Profile of Traditionally Used Medicinal Plant Hyssop (*Hyssopus Officinalis* L.). J. Appl. Pharm. Sci..

[223] Darzi M.T., Nekoo B.S. (2016). Effects of Organic Amendments and Biofertilizer Application on Some Morphological Traits and Yield of Hyssop (*Hyssopus Officinalis* L.). J. Hortic. Sci..

[224] Sharifi P. (2017). The Effect of Plant Growth Promoting Rhizobacteria (PGPR), Salicylic Acid and Drought Stress on Growth Indices, the Chlorophyll and Essential Oil of Hyssop (*Hyssopus Officinalis*). Biosci. Biotechnol. Res. Asia.

[225] Xiong Y.W., Li X.W., Wang T.T., Gong Y., Zhang C.M., Xing K., Qin S. (2020). Root Exudates-Driven Rhizosphere Recruitment of the Plant Growth-Promoting Rhizobacterium *Bacillus Flexus* KLBMP 4941 and Its Growth-Promoting Effect on the Coastal Halophyte *Limonium Sinense* under Salt Stress. Ecotoxicol. Environ. Saf..

[226] Barnawal D., Pandey S.S., Bharti N., Pandey A., Ray T., Singh S., Chanotiya C.S., Kalra A. (2017). ACC Deaminase-Containing Plant Growth-Promoting Rhizobacteria Protect *Papaver Somniferum* from Downy Mildew. J. Appl. Microbiol..

[227] Asghari B., Khademian R., Sedaghati B. (2020). Plant Growth Promoting Rhizobacteria (PGPR) Confer Drought Resistance and Stimulate Biosynthesis of Secondary Metabolites in Pennyroyal (*Mentha Pulegium* L.) under Water Shortage Condition. Sci. Hortic..

[228] El-Serafy R.S., El-Sheshtawy A.A. (2020). Effect of Nitrogen Fixing Bacteria and Moringa Leaf Extract on Fruit Yield, Estragole Content and Total Phenols of Organic Fennel. Sci. Hortic..

[229] Ghanbarzadeh Z., Mohsenzadeh S., Rowshan V., Zarei M. (2020). Mitigation of Water Deficit Stress in Dracocephalum Moldavica by Symbiotic Association with Soil Microorganisms. Sci. Hortic..

[230] Kang J.P., Huo Y., Yang D.U., Yang D.C. (2020). Influence of the Plant Growth Promoting Rhizobium Panacihumi on Aluminum Resistance in Panax Ginseng. J. Ginseng Res..

[231] Huo Y., Kang J.P., Ahn J.C., Kim Y.J., Piao C.H., Yang D.U., Yang D.C. (2021). Siderophore-Producing Rhizobacteria Reduce Heavy Metal-Induced Oxidative Stress in Panax Ginseng Meyer. J. Ginseng Res..

[232] Chiappero J., del Rosario Cappellari L., Sosa Alderete L.G., Palermo T.B., Banchio E. (2019). Plant Growth Promoting Rhizobacteria Improve the Antioxidant Status in *Mentha Piperita* Grown under Drought Stress Leading to an Enhancement of Plant Growth and Total Phenolic Content. Ind. Crops Prod..

[233] Del Rosario Cappellari L., Santoro M.V., Schmidt A., Gershenzon J., Banchio E. (2019). Induction of Essential Oil Production in *Mentha* x *Piperita* by Plant Growth Promoting Bacteria Was Correlated with an Increase in Jasmonate and Salicylate Levels and a Higher Density of Glandular Trichomes. Plant Physiol. Biochem..

[234] Seif Sahandi M., Mehrafarin A., Naghdi Badi H., Khalighi-Sigaroodi F., Sharifi M. (2019). Improving Growth, Phytochemical, and Antioxidant Characteristics of Peppermint by Phosphate-Solubilizing Bacteria along with Reducing Phosphorus Fertilizer Use. Ind. Crops Prod..

[235] Singh S., Tripathi A., Maji D., Awasthi A., Vajpayee P., Kalra A. (2019). Evaluating the Potential of Combined Inoculation of *Trichoderma Harzianum* and *Brevibacterium Halotolerans* for Increased Growth and Oil Yield in *Mentha Arvensis* under Greenhouse and Field Conditions. Ind. Crops Prod..

[236] Chandra S., Askari K., Kumari M. (2018). Optimization of Indole Acetic Acid Production by Isolated Bacteria from Stevia Rebaudiana Rhizosphere and Its Effects on Plant Growth. J. Genet. Eng. Biotechnol..

[237] Del Rosario Cappellari L., Chiappero J., Santoro M.V., Giordano W., Banchio E. (2017). Inducing Phenolic Production and Volatile Organic Compounds Emission by Inoculating *Mentha Piperita* with Plant Growth-Promoting Rhizobacteria. Sci. Hortic..

[238] Tahami M.K., Jahan M., Khalilzadeh H., Mehdizadeh M. (2017). Plant Growth Promoting Rhizobacteria in an Ecological Cropping System: A Study on Basil (*Ocimum Basilicum* L.) Essential Oil Production. Ind. Crops Prod..

[239] Jha Y., Subramanian R.B. (2016). Rhizobacteria Enhance Oil Content and Physiological Status of Hyptis Suaveolens under Salinity Stress. Rhizosphere.

[240] Llorente B.E., Alasia M.A., Larraburu E.E. (2016). Biofertilization with Azospirillum Brasilense Improves in Vitro Culture of Handroanthus Ochraceus, a Forestry, Ornamental and Medicinal Plant. New Biotechnol..

[241] Zhao Q., Wu Y.N., Fan Q., Han Q.Q., Paré P.W., Xu R., Wang Y.Q., Wang S.M., Zhang J.L. (2016). Improved Growth and Metabolite Accumulation in Codonopsis Pilosula (Franch.) Nannf. by Inoculation of Bacillus Amyloliquefaciens GB03. J. Agric. Food Chem..

[242] Ogbe A.A., Finnie J.F., Van Staden J. (2020). The Role of Endophytes in Secondary Metabolites Accumulation in Medicinal Plants under Abiotic Stress. S. Afr. J. Bot..

[243] Guo J., Muhammad H., Lv X., Wei T., Ren X., Jia H., Atif S., Hua L. (2020). Prospects and Applications of Plant Growth Promoting Rhizobacteria to Mitigate Soil Metal Contamination: A Review. Chemosphere.

[244] Gouvea D.R., Gobbo-Neto L., Lopes N.P. (2012). The Influence of Biotic and Abiotic Factors on the Production of Secondary Metabolites in Medicinal Plants. Plant Bioact. Drug Discov. Princ. Pract. Perspect..

[245] Shahzad S.M., Arif M.S., Ashraf M., Abid M., Ghazanfar M.U., Riaz M., Yasmeen T., Zahid M.A. (2015). Alleviation of Abiotic Stress in Medicinal Plants by PGPR. Plant-Growth-Promoting Rhizobacteria (PGPR) and Medicinal Plants.

[246] Lim J.-H., Kim S.-D. (2013). Induction of Drought Stress Resistance by Multi-Functional PGPR Bacillus Licheniformis K11 in Pepper. Plant Pathol. J..

[247] Heidari M., Mousavinik S.M., Golpayegani A. (2011). Plant Growth Promoting Rhizobacteria (PGPR) Effect on Physiological Parameters and Mineral Uptake in Basil (*Ociumum Basilicm* L.) under Water Stress. J. Agric. Biol. Sci..

[248] Ghorbanpour M., Hatami M., Khavazi K. (2013). Role of Plant Growth Promoting Rhizobacteria on Antioxidant Enzyme Activities and Tropane Alkaloid Production of Hyoscyamus Niger under Water Deficit Stress. Turk. J. Biol..

[249] Xu Z., Zhou J., Ren T., Du H., Liu H., Li Y., Zhang C. (2020). Salt Stress Decreases Seedling Growth and Development but Increases Quercetin and Kaempferol Content in *Apocynum venetum*. Plant Biol..

[250] Banerjee A., Roychoudhury A. (2017). Effect of Salinity Stress on Growth and Physiology of Medicinal Plants. Medicinal Plants and Environmental Challenges.

[251] Chauhan N., Kumar D. (2014). Effect of Salinity Stress on Growth Performance of Citronella Java. Int. J Geol. Agric. Environ. Sci..

[252] Mondal H.K., Kaur H. (2017). Effect of Salt Stress on Medicinal Plants and Its Amelioration by Plant Growth Promoting Microbes. Int. J. Bio-Resour. Stress Manag..

[253] Rabiei Z., Hosseini S.J., Pirdashti H., Hazrati S. (2020). Physiological and Biochemical Traits in Coriander Affected by Plant Growth-Promoting Rhizobacteria under Salt Stress. Heliyon.

[254] Bharti N., Barnawal D., Awasthi A., Yadav A., Kalra A. (2014). Plant Growth Promoting Rhizobacteria Alleviate Salinity Induced Negative Effects on Growth, Oil Content and Physiological Status in Mentha Arvensis. Acta Physiol. Plant..

[255] Egamberdieva D., Jabborova D., Mamadalieva N. (2013). Salt Tolerant Pseudomonas Extremorientalis Able to Stimulate Growth of Silybum Marianum under Salt Stress. Med. Aromat. Plant. Sci. Biotechnol..

[256] Egamberdieva D. (2012). Pseudomonas Chlororaphis: A Salt-Tolerant Bacterial Inoculant for Plant Growth Stimulation under Saline Soil Conditions. Acta Physiol. Plant..

[257] Danish S., Kiran S., Fahad S., Ahmad N., Ali M.A., Tahir F.A., Rasheed M.K., Shahzad K., Li X., Wang D. (2019). Alleviation of Chromium Toxicity in Maize by Fe Fortification and Chromium Tolerant ACC Deaminase Producing Plant Growth Promoting Rhizobacteria. Ecotoxicol. Environ. Saf..

